# An Overview of Modeling Approaches for Compositional Control in III–V Ternary Nanowires

**DOI:** 10.3390/nano13101659

**Published:** 2023-05-17

**Authors:** Egor D. Leshchenko, Vladimir G. Dubrovskii

**Affiliations:** Faculty of Physics, St. Petersburg State University, Universitetskaya Emb. 13B, 199034 St. Petersburg, Russia; dubrovskii@mail.ioffe.ru

**Keywords:** III–V ternary nanowires, composition, modeling, vapor–liquid–solid mechanism, growth kinetics

## Abstract

Modeling of the growth process is required for the synthesis of III–V ternary nanowires with controllable composition. Consequently, new theoretical approaches for the description of epitaxial growth and the related chemical composition of III–V ternary nanowires based on group III or group V intermix were recently developed. In this review, we present and discuss existing modeling strategies for the stationary compositions of III–V ternary nanowires and try to systematize and link them in a general perspective. In particular, we divide the existing approaches into models that focus on the liquid–solid incorporation mechanisms in vapor–liquid–solid nanowires (equilibrium, nucleation-limited, and kinetic models treating the growth of solid from liquid) and models that provide the vapor–solid distributions (empirical, transport-limited, reaction-limited, and kinetic models treating the growth of solid from vapor). We describe the basic ideas underlying the existing models and analyze the similarities and differences between them, as well as the limitations and key factors influencing the stationary compositions of III–V nanowires versus the growth method. Overall, this review provides a basis for choosing a modeling approach that is most appropriate for a particular material system and epitaxy technique and that underlines the achieved level of the compositional modeling of III–V ternary nanowires and the remaining gaps that require further studies.

## 1. Introduction

**Figure 1:**
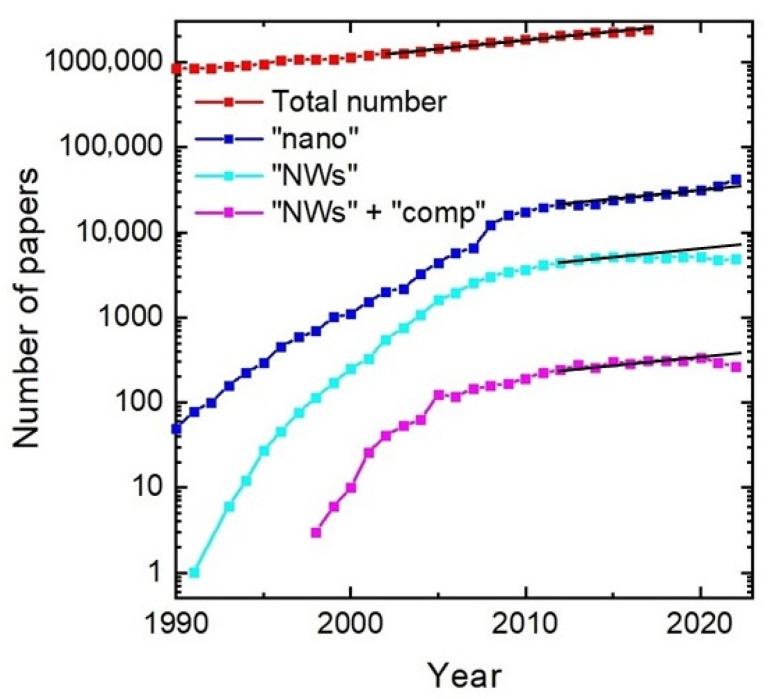
Number of papers published per year. The total number of papers is taken from Ref. [30]. The blue, cyan, and magenta curves are obtained by the Scopus search using the keywords “nano”, “nanowire”, and “nanowire + composition”, respectively. The black line corresponds to the growth rate of the total number of papers.

Among semiconductor nanostructures of various shapes and dimensions [1,2], III–V nanowires [3,4,5] and heterostructures based on such nanowires [6,7,8,9] are some of the most promising building blocks for fundamental research in nanoscience and technological applications [10,11,12]. This is due to the possibility of dislocation-free growth of III–V nanowires on silicon substrates [13] and an almost unlimited flexibility in tuning the nanowire length [14], radius [15], chemical composition [16], position [17], surface density [18], crystal structure [19,20], interface properties across a heterostructure [21], and doping levels [22]. The first whisker-like Si crystals with a radius of down to 100 nm were grown via the Au-catalyzed vapor–liquid–solid (VLS) mechanism by Wagner and Ellis in 1964 [23]. Later on, nanowire research was conducted by Givargizov [24,25] and Sakaki [26], but overall interest in the topic somehow declined. A renaissance at the true nanoscale started in the late 1990s, mostly by the research groups of Lieber [27], Samuelson [28], and Yang [29]. The growing interest in the nanowire field is reflected in the annual growth rate of the number of papers related to nanowires, which for a long time (starting from the late 1990s to 2005) exceeded the growth rate of the total number of papers and even the total number of papers in the field of nanoscience (see Figure 1).

Despite 30 years of research and a large number of nanowire-based device structures, including solar cells [31,32], photodetectors [33], biosensors [34], transistors [35], resonant tunnelling diodes [36], lasers [37], and piezoelectric nanogenerators [38], semiconductor nanowires face challenges in their implementation in the manufacturing industry. To be competitive in a marketplace, new technology should be either cheaper and simpler than the existing one or provide otherwise unattainable functionalities. The common techniques to fabricate high-quality nanowires [10,39] require expensive substrates, sources of pure elements, and equipment (especially if one uses lithography for the substrate preparation), which excludes the first “low-cost” way. However, attaining new functionalities is almost unlimited using III–V ternary nanowires and their heterostructures, whose composition and coherent growth on Si substrates is not restricted by the lattice mismatch. Considering ternary nanowires, the ratio x between A and B elements (or AD and BD pairs) in solid A_x_B_1−x_D material, called the solid composition, determines the nanowire optoelectronic properties, including the band gap [40,41]. This explains the importance of studying compositional control in ternary nanowires and the general shift in research focus from binary (GaAs [42], GaN [43], InAs [42]) to ternary III–V (InGaAs, InGaN, AlGaAs) and even quaternary nanowires. Figure 1 shows that, after explosive growth, research interest in nanoscience and nanotechnology, especially in nanowires, fell quite drastically. As a result, the growth rate of nanotechnology-related papers became equal to the total growth rate of all papers, while the nanowire topic in general has passed its maximum and is in decline at the moment. However, research on ternary nanowires remains a hot topic.

The introduction of a liquid droplet at the growth interface of VLS III–V nanowires brings new possibilities for tailoring the morphology, crystal phase, and composition of nanowires, but it largely complicates the growth process of ternary nanowires. As a result, the vapor–solid distributions of VLS NWs and two-dimensional (2D) epitaxial layers grown under the same conditions are different. For example, the incorporation of Sb into InAs_1−x_Sb_x_ nanowires is significantly higher than that into 2D layers obtained in the same growth run [44]. Depending on the growth conditions, different processes may limit the growth process and hence play a key role in the resulting nanowire composition. In general, epitaxial growth can be limited by thermodynamics at high temperatures and chemical reaction rates at low temperatures. Intermediate temperatures should correspond to the surface-transport-limited growth regimes, where surface diffusion of group III adatoms becomes very important. These limiting steps are well understood for thin films [45], but they should also be relevant for nanowires [46,47]. Different limiting steps of the mass transport mechanisms resulted in the development of different modeling approaches for ternary III–V nanowire compositions, which are based on different assumptions. In this review, we describe the existing models, discuss their advantages and drawbacks, reveal the key parameters and factors influencing the nanowire growth mechanisms, and analyze their impact on the composition of ternary III–V nanowires.

## 2. Experimental Works

Compositions of III–V ternary nanowires based on group III (III_x_III_1−x_–V) and group V (III–V_x_V_1−x_) intermix, grown via the VLS or the catalyst-free vapor–solid (VS) mechanisms, were experimentally studied in more than 80 papers. An overview can be found, for example, in Refs. [48,49]. The compositions of GaAsSb [50], InAsSb [44], InGaAs [16], InGaP [51], AlGaAs [52], and InGaN [53] NWs have been studied in much detail, while there is a lack of knowledge on compositional control in GaSbP [54], InSbP [55], AlInP [56], and AlGaP [57] materials systems. The composition of most ternary nanowires (including materials systems with a strong interaction between AD and BD pairs, such as InGaAs and InGaN) can be varied over almost the entire compositional range. This is in sharp contrast with thin films, where a combination of the lattice-mismatched materials leads to miscibility gaps and the segregation of pure binaries [58]. There are some unresolved problems with growing Sb-based nanowires, such as InAs_1−x_Sb_x_ [59] and GaSb_x_P_1−x_ [54]. These nanowires show non-uniform morphology, strong tapering, and a narrow range of composition tuning (of less than 15% in the Sb content). An increase in Sb flux usually leads to a higher radial growth rate and two-dimensional growth of GaAs_1−x_ Sb_x_ [60]. Nanowires with high Sb content can be grown on InAs or GaAs stems [44,60]. In InAs_1−x_Sb_x_, the stacking fault density monotonically decreases [59,61], while the density of twin defects increases [59] with Sb content. Wurtzite-zincblende (WZ-ZB) polytypism generally plays an important role in the structure of III–V nanowires and may depend on the nanowire composition [62,63]. In particular, InAs_1−x_Sb_x_ nanowires tend to form in the WZ phase at low Sb content (x≈2%) and in the ZB phase at high Sb content (x≈10%) [59,61].

Nanowire growth involves different homogeneous and heterogeneous reactions in the growth chamber, transport of the precursors in metal organic vapor-phase epitaxy (MOVPE [64,65]), hydride vapor-phase epitaxy with chloride precursors of group III species (HVPE [66]), or group III atoms and group V molecules in molecular beam epitaxy (MBE [67]), elementary processes at the substrate and nanowire surfaces (impingement, surface diffusion, re-emission), and atom incorporation into the solid phase of a nanowire. The composition of a III–V ternary nanowire is influenced by many factors, including the following.

− Material system which defines the difference in the chemical potentials for pure elements and the shape of liquid–solid composition dependence in the case of VLS nanowires. For example, the composition of VLS In_x_Ga_1−x_As nanowires cannot be understood without accounting for the predominance of liquid in in the catalyst droplet [68].
− Growth method and equipment that determine the transfer of the precursors (or atoms).
− Size distribution of the initial droplets [46,69] and the resulting radii of VLS nanowires. This has an effect on the effective flux of atoms that feed the droplet [70,71,72,73].
− Pitch dependence of the nanowire growth rates [74]. Decreasing the pitch leads to a larger competition of the neighboring nanowires for the arriving growth species [71,75].
− Surface temperature during growth [46,76,77] is one of the most complex parameters because it simultaneously influences the pyrolysis efficiencies in VPE techniques [45], surface diffusion for VS nanowires [46], binary and ternary interactions in the droplet for VLS nanowires [78], evaporation rates from the substrate surface or droplets [70], and attachment and detachment rates of a ternary island.
− The flux ratio of A to B atoms in vapor is the main control parameter that influences the composition of a ternary nanowire. Higher vapor flux of one of the elements is expected to yield its higher content in solids. The flux ratio influences the nanowire growth kinetics, shadowing effect [79], and elementary processes, such as the direct impingement, diffusion from the substrate and nanowire sidewalls to its top, and evaporation [70]. In the case of growth on the reflecting masked substrates such as SiO_x_/Si, the situation becomes even more complex. In the initial growth, a nanowire ensemble consumes only a part of the reflected flux [71,72,73]. Long enough nanowires consume the entire group III fluxes sent from the vapor. However, the saturation lengths may be different for A and B species and depend on the A/B flux ratio.
− The total III/V flux ratio may enhance or suppress the incorporation of one of the elements (A or B) into solid nanowires even at a fixed A/B ratio. For example, the content of GaSb in In_x_Ga_1−x_Sb nanowires decreases with an increase in the TMSb molar fraction [80].
− The type of growth catalyst for VLS NWs generally influences the binary supersaturation values [78,81]. Furthermore, the composition of different growth constituencies in liquid is generally different from their vapor contents, particularly for highly volatile group V molecules, such as As_2_, P_2_, or N_2_. Au remains one of the most common catalysts for VLS NW growth [82,83]. However, it might lead to unwanted nanowire contamination [84,85]. This issue is safely avoided in self-catalyzed VLS growth [86,87], where the foreign Au catalyst is replaced by one of the nanowire constituents (a group III element, such as Ga or In). This growth technique is very promising for the fine tuning of the nanowire morphology by changing the droplet volume under a varying III/V flux ratio, radius self-equilibration effect, sharpening the nanowire tips, etc. [15,88,89].
− Group V concentration in the catalyst droplets can be changed by varying the group V flux or III/V flux ratio. Unfortunately, its typical values (on the order of 1%) are lower than the detection limit of any of the characterization techniques, including energy-dispersive X-ray spectroscopy. On the other hand, the group V concentration is known to have a tremendous effect on the supersaturation [81,90].

## 3. General Remarks and Definitions

The cluster approach is the core of most models for ternary nanowire composition. Here, the “cluster” may be a small 2D nucleus of a III–V ternary nanowire which nucleates at the vapor–solid or liquid–solid interface, or a larger 2D island or fractional monolayer of a nanowire which grows by attaching and detaching AD and BD pairs. This approach assumes the existence of a distinct interface between a growing cluster and a mother phase. Two important questions arise immediately: (1) where is the dividing surface that separates the cluster and the mother phase; and (2) what is the surface energy of the interface? Within the capillarity approximation [91,92,93], clusters are treated as macroscopic objects. The surface energy of a cluster is then described in terms of macroscopic interfacial tension. In this approximation, the surface energy of a small circular 2D cluster is the same as the surface energy of an infinitely large layer. Clearly, this approximation becomes inaccurate for very small clusters (which may consist of only a few atoms or III–V pairs) and may result in overestimated nucleation rates [94]. In the alternative density-functional approach [95], a continuous change in molecular number density through a transition zone is considered instead of the dividing surface. Thus, the distinction between the bulk and surface atoms or molecules disappears. The solution to the corresponding variational problem is not possible in the general case, and one has to rely on some approximations. The gradient, hard-sphere, and quasi-thermodynamic approximations are generally considered. These approximations limit the application of the models based on the density-functional approach. The situation occurs even for complex for III–V ternary materials.

Generally, the density-functional theory is a computational quantum mechanical method used to calculate the nuclear (or, electronic) structure of many-body systems. It belongs to the family of first principles methods and could be used to explain the experimental results from the nanoscale scope. The basic concepts and practical details can be found in Refs. [96,97,98]. In the field of nanowires, the density-functional theory within the local density and generalized gradient approximations was used to describe the Au-catalyzed and self-catalyzed growth of GaAs nanowires under near-equilibrium conditions [99]. In particular, it has been shown that the droplet on the nanowire tip has a contact angle of around $130^{o}$, in agreement with experimental observations [100]. The catalytic effect of the Au droplet on GaAs nanowire growth was considered in Ref. [101]. To the best of our knowledge, there are no models based on the density-functional approach that describe the composition of ternary nanowires. Therefore, this review focuses on the first approach of treating III–V islands with distinct boundary.

Let us introduce the solid, vapor, and liquid compositions in the most general case of ternary A_x_B_1−x_D nanowires growing from a liquid droplet resting at the nanowire top [68]. Nanowire composition is determined by the content of AD pairs in the nanowire or, more precisely, in a growing fractional monolayer as follows:

(1) $$x=\frac{N_{AD}}{N_{AD}+N_{BD}},$$

where $N_{AD}$ and $N_{BD}$ are the numbers of AD and BD pairs in the nanowire, respectively.

The vapor phase is fully characterized by the atomic fluxes of A, B, and D elements ($I_{A}$, $I_{B}$, and $I_{D}$, respectively). The ratio

(2) $$z=\frac{I_{A}}{I_{A}+I_{B}}$$

is called the vapor composition. Taking into account different kinetic pathways for the material currents that feed the droplet (the direct impingement, re-emission, and diffusion from the substrate surface and nanowire sidewalls [70]), the relative influx of element A entering the droplet can be written as follows:

(3) $$Z=\frac{V_{A}}{V_{A}+V_{B}}.$$

Here, $V_{A}$ and $V_{B}$ are the atomic influxes which include the geometrical effects and the diffusion-induced contributions. For example, for short enough nanowires, the atomic influx of a group III element i can be presented as $V_{i}=I_{i}\left(\chi_{i}^{+}+\varphi_{i}\lambda_{fi}/R+\varphi_{i}{\left(\lambda_{si}/R\right)}^{2}\right)$, where $\lambda_{fi}$ and $\lambda_{si}$ are the diffusion lengths on the sidewalls and substrate, respectively. R is the nanowire radius and $\chi_{i}^{+}$, $\varphi_{i}$ are the coefficients that describe geometrical effects and precursor cracking efficiencies at the surface and nanowire surfaces [18,102]. Then, the relationship between Z and z is given as follows:

(4) $$Z=\frac{z}{z+\gamma \left(1-z\right)},$$

with

(5) $$\gamma =\frac{\chi_{B}^{+}+\varphi_{B}\lambda_{fB}/R+\varphi_{B}{\left(\lambda_{sB}/R\right)}^{2}}{\chi_{A}^{+}+\varphi_{A}\lambda_{fA}/R+\varphi_{A}{\left(\lambda_{sA}/R\right)}^{2}}$$

for short nanowires and

(6) $$\gamma =\frac{\chi_{B}^{+}+\varphi_{B}\lambda_{fB}/R}{\chi_{A}^{+}+\varphi_{A}\lambda_{fA}/R}$$

for long nanowires.

The composition of a quaternary A_y_B_1−y_DU liquid droplets is characterized by the concentrations of A ($c_{A}$), B ($c_{B}$), D ($c_{D}$), and U ($c_{U}$) elements. The element U denotes a foreign catalyst, such as Au. The A content in liquid is defined as the following:

(7) $$y=\frac{c_{A}}{c_{A}+c_{B}}.$$

In self-catalyzed VLS growth, the concentration of a foreign catalyst metal becomes zero ($c_{U}=0$). Usually, the total concentration of A and B atoms is denoted as $c_{tot}=c_{A}+c_{B}$. Because the concentrations obey the normalization condition ($c_{A}+c_{B}+c_{D}+c_{U}=1$), there is flexibility in the choice of the three independent variables. For example, the composition of a quaternary droplet is fully characterized by the $c_{D}$, $c_{U}$, and y variables.

The main goal of the theory is to describe the formation process of a ternary III–V nanowire, which provides a relationship between the solid and vapor compositions x(z) (the vapor–solid distribution), or at least between the solid and liquid compositions x(y) (the liquid–solid distribution). Depending on the distribution type provided by a model, we divide the existing approaches into the models describing the liquid–solid incorporation mechanisms (the equilibrium, nucleation, and kinetic models treating the growth of solid from liquid) and those describing the vapor–(liquid)–solid incorporation mechanisms and finally yield vapor–solid distribution (empirical, material balance, reaction-based models, and kinetic models treating the growth of solid from vapor).

## 4. Liquid–Solid Incorporation Models

**Figure 2:**
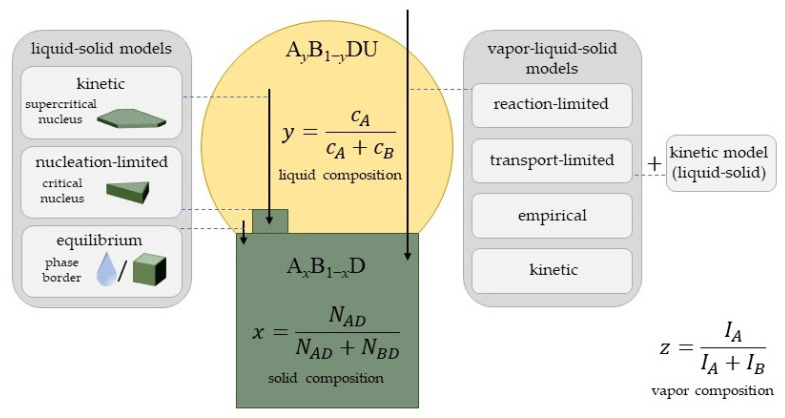
Scheme of different modeling strategies in the liquid–solid (**left**) and vapor–liquid–solid or vapor–solid (**right**) incorporation models.

These models describe the incorporation mechanisms of different atoms entering the solid from a liquid droplet, while the material exchange between the droplet and vapor [70] is not considered. The droplet composition should be exactly known to access the solid composition. This is a general drawback of all the models treating the liquid–solid growth without taking into account the vapor phase for several reasons. First, the concentration of group V elements in the droplet is usually too low to be experimentally detected, while it influences the calculated composition very significantly. Second, measurements of the liquid composition after growth may be altered by the droplet consumption under group V flux or the cooling-down process (it becomes more reliable if one uses in situ techniques, such as growth in environmental transmission electron microscopes (ETEMs) [103,104,105]). Third, the real control parameters of any growth process are the material fluxes at a given temperature rather than the liquid composition, although the vapor composition influences the liquid state. The equilibrium, nucleation-limited, and kinetic liquid–solid incorporation models are schematized in Figure 2. There are some modifications of the models that allow one to link the vapor and solid compositions. For example, an interesting combination of the kinetic model with the material balance equations which does not require any fitting parameters has been used to explain the growth and composition of Al_x_Ga_1−x_P nanowires [106]. However, the main focus of the liquid–solid incorporation models is on the liquid–solid distribution x(y).

### 4.1. Equilibrium Models

The core idea of this approach is that the growth process of a ternary material occurs under close-to-equilibrium conditions between the vapor (for VS growth) or liquid (for VLS growth) and solid phases. Then, the growth species are studied under equilibrium conditions between the two phases [21]; however, strictly speaking, thermodynamic equilibrium corresponds to no-growth conditions. Thermodynamic equilibrium implies thermal (no net flow of thermal energy), mechanical (the pressures of the two phases are equal), and chemical (the reaction rates of the direct and reversed reactions equal each other) equilibria. Because nanowires grow at a fixed temperature and pressure, the governing equation that describes the ternary composition is given by zero chemical potential difference between the two phases (a mixture of gases in the VS process and ternary or even quaternary liquid alloy in the catalyst droplet for VLS growth). Taking an example of the VLS process and considering equilibrium liquid (l) and solid (s) phases, we have $\Delta \mu \equiv \mu^{l}-\mu^{s}=0$.

**Figure 3:**
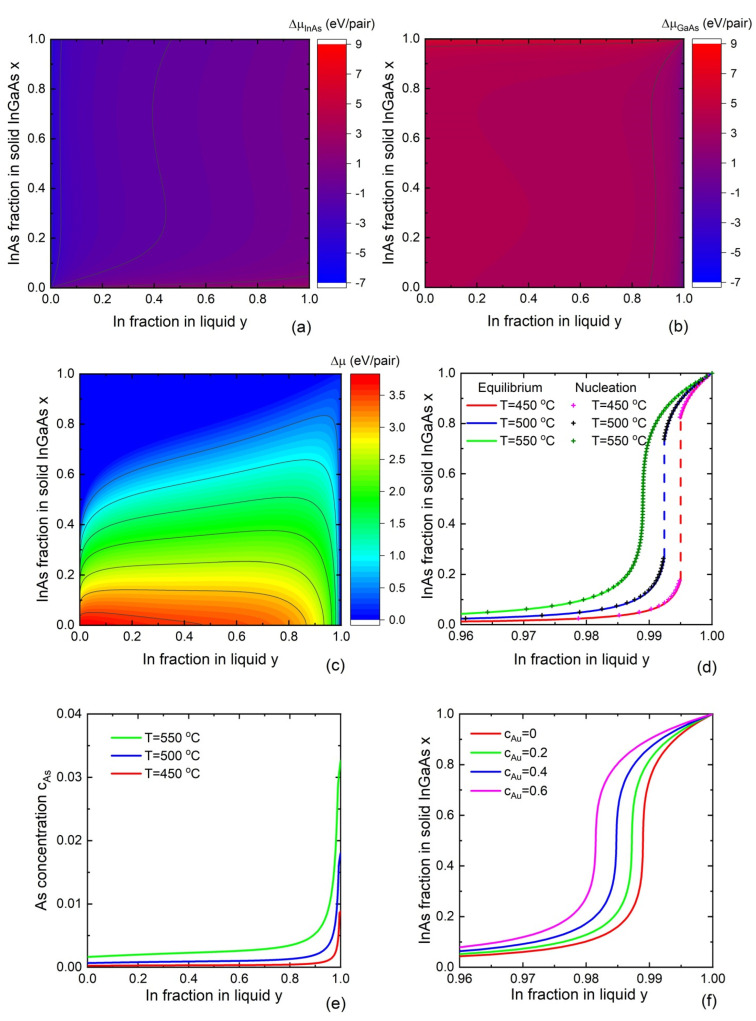
Chemical potential differences between the liquid and solid (**a**) InAs and (**b**) GaAs binaries at a fixed $c_{Au}=0$, $c_{As}=0.01$, and T=450 °C. (**c**) Contour map of the chemical potential difference for ternary InGaAs as a function of the liquid and solid In content at a fixed $c_{Au}=0$, $c_{As}=0.01$, and T=450 °C, obtained from the general equilibrium condition. (**d**) Liquid–solid distributions of self-catalyzed In_x_Ga_1−x_As nanowires x(y) and (**e**) As concentrations obtained from Equations (9) and (10) (solid curves) at different temperatures, from 450 °C to 550 °C. Symbols show the nucleation-limited liquid–solid distributions given by Equation (17) at a fixed $c_{As}=0.01$. Vertical dashed lines correspond to the miscibility gaps below the critical temperature of 543 °C. (**f**) Liquid–solid distributions of Au-catalyzed In_x_Ga_1−x_As nanowires obtained from Equations (9) and (10) at a fixed T=550 °C and different Au concentrations shown in the legend.

In *the general case,* thermodynamic equilibrium of a ternary A_x_B_1−x_D nanowire with a liquid droplet is given as follows:

(8) $$x\Delta \mu_{AD}+\left(1-x\right)\Delta \mu_{BD}=0,$$

where $\Delta \mu_{AD}$ and $\Delta \mu_{BD}$ are the differences of chemical potential for AD and BD pairs in the two phases in thermal units. From Equation (8), supersaturation of one binary ($\Delta \mu_{AD}>0$) requires undersaturation of the other binary ($\Delta \mu_{BD}<0$) [95]. Figure 3a,b shows the chemical potential differences for InAs and GaAs pairs calculated at a fixed T=450 °C, $c_{As}=0.01$, and $c_{Au}=0$ (corresponding to self-catalyzed VLS growth) using the parameters given in the Supporting Information (SI). Figure 3c shows the contour maps of the chemical potential difference between the liquid and solid InGaAs ternaries as a function of the liquid and solid compositions y and x. Formation of solid InGaAs is possible within the colored area of the compositional map and is forbidden within the blue area. Therefore, this model separates the range of liquid and solid compositions that are accessible or inaccessible, respectively, under equilibrium. To our knowledge, the model based on the general equilibrium condition has not been applied for the description of ternary nanowire composition so far.

The chemical potential of species i=(A,B,D) in the liquid phase can be expressed as $\mu_{i}^{L}=\mu_{i}^{0}+\ln\left(c_{i}\right)+\psi_{i}$. The first term is the chemical potential of pure liquids. The second term is the configuration entropy of mixing. For the interaction term $\psi_{i}$, we use the regular solution model [107], with the interaction parameters given by Redlich–Kister polynomials [108]. The exact form of $\psi_{i}$ is given in the Supporting Information (SI). The chemical potentials of the AD and BD pairs in solids are given by $\mu_{AD}^{s}=\mu_{AD}^{0}+\ln x+\omega_{s}{\left(1-x\right)}^{2}$ and $\mu_{BD}^{s}=\mu_{BD}^{0}+\ln\left(1-x\right)+\omega_{s}x^{2}$, respectively, with $\mu_{AD}^{0}$ and $\mu_{BD}^{0}$ as the chemical potentials of AD and BD solid binaries. The pseudo-binary interaction parameter $\omega_{s}$ can be expressed through electronegativities and solubility parameters of pure components [45] or obtained by thermodynamic assessment using the CALPHAD method [109]. The values of the chemical potentials of pure liquids and solids, as well as the different interaction parameters used for calculations, are given in the Supporting Information (SI), Tables S1–S5.

If the binary chemical potentials are *decoupled*, thermodynamic equilibrium for the AD and BD pairs in the liquid and solid state is given by [21]:

(9) $$\Delta \mu_{AD}=0,$$

(10) $$\Delta \mu_{BD}=0.$$

Obviously, this is a particular case of the general equilibrium condition. In this case, the liquid–solid distribution can be presented in the following form:

(11) $$y=\frac{x}{x+\left(1-x\right)e^{2\omega_{S}\left(x-1/2\right)+b}},$$

while the concentration of D atoms equals

(12) $$c_{D}=\frac{x}{y}\frac{1}{c_{tot}}e^{\omega_{S}{\left(1-x\right)}^{2}+b_{D}}.$$

Here, $\omega_{S}$ is the pseudo-binary interaction parameter of the AD and BD pairs in solids, and b and $b_{D}$ are y-dependent parameters whose form can be found in the Supporting Information (SI).

For self-catalyzed nanowires, the VLS system is described by the solid composition x and the two concentrations of D and A atoms in liquid. Under equilibrium conditions, the liquid phase is described by only one variable, namely the A content in the droplet y. The solid compositions of self-catalyzed In_x_Ga_1−x_As nanowires and As concentrations versus the In content in liquid at different temperatures are shown in Figure 3d,e, respectively. For simplicity, Equations (11) and (12) are solved ignoring the ternary and composition-dependent binary interaction parameters in liquid. According to the equilibrium model, synthesis of In_x_Ga_1−x_As nanowires with a significant InAs fraction x>0.1 requires very high In/Ga ratios in liquid, corresponding to y>0.98. The InAs fraction in solid increases with temperature at a fixed In concentration in liquid. The As concentration in liquid increases with temperature and the In content in liquid. Its value remains very low, which is well known for nanowire growth via the VLS mechanism. As expected, the miscibility gap (corresponding to the dashed vertical lines in the figures), which depends only on temperature, shrinks with temperature and disappears at the critical temperature 𝑇 = 543 °C, corresponding to $\omega_{S}=2$. Because the equilibrium model predicts the liquid–solid distribution, which is very close to the one obtained from the nucleation-limited model, a theoretical explanation of the temperature dependence of the solid composition is given in the next section.

To our knowledge, the influence of the concentration of a foreign catalyst on liquid–solid distribution shapes has not been studied within the equilibrium model. We consider this dependence in the case of the quaternary Au-In-Ga-As droplet, where the Au concentration in liquid becomes the second independent variable. Figure 3f shows the solid composition of In_x_Ga_1−x_As as a function of the liquid composition y, calculated for different Au concentrations of $c_{Au}=0,\;0.2,\;0.4$, and 0.6. It is seen that increasing the Au concentration in the droplet leads to an increase in InAs fraction in InGaAs nanowires at a fixed liquid composition.

The equilibrium model based on Equations (9) and (10) has been extensively used for the description of VS growth [45]. As for III–V ternary nanowires, the model provided good fits to the experimental compositional profiles across axial GaAs/Al_x_Ga_1−x_As/GaAs nanowire heterostructures [21]. It has been shown that the liquid–solid distribution for this lattice-matched system with $\omega_{S}≅0$ is similar to the Langmuir–McLean formula for a segregating system [110]. In Ref. [111], the equilibrium and nucleation-limited models were compared in the case of self-catalyzed Al_x_Ga_1−x_As nanowires and axial heterostructures based on such nanowires. In particular, the effect of As concentration on the liquid–solid distribution has been studied using both models with very similar results.

The main advantage of the decoupled equilibrium model for AB and BD binaries grown from liquid is the absence of any free parameters in the case of self-catalyzed VLS growth. The liquid–solid distribution in this case is fully determined by the material parameters and temperature. In the case of Au-catalyzed VLS growth, one should consider the Au concentration in the droplet, which influences the liquid–solid distribution. Introduction of Au is entirely possible because its concentration can reliably be measured after growth. Therefore, it is possible to check if a ternary nanowire forms under equilibrium conditions for the liquid and solid phases. On the other hand, considering only the liquid–solid equilibrium does not provide any relationship between the solid and vapor compositions (which is a general drawback of all liquid–solid incorporation models, as mentioned above). For example, the influence of the V/III flux ratio on the solid composition [44] can be accounted for only if the equilibrium model is applied to both the vapor and solid phases. Overall, the equilibrium model should provide a limit for the solid composition because it corresponds to a very slow growth process in which the chemical potential difference approaches zero.

### 4.2. Nucleation-Limited Model

It is well documented that droplet-seeded VLS nanowires grow in the layer-by-layer mononuclear regime, in which each nanowire monolayer forms from a single nucleus or 2D island. The next nucleation event occurs only upon completion of the preceding monolayer, as confirmed by in situ studies of VLS GaAs nanowires in ETEM [112,113]. The nucleation-limited model describes the formation of a ternary III nucleus of a critical size, above which the island tends to grow at a given supersaturation of liquid. The nucleus formation energy as a function of the number of III–V pairs s includes two terms of different signs. The energy released in the liquid–solid transition is positive and proportional to s. The surfaces, or interface energy of the island, are negative and are proportional to the nucleus perimeter $\sqrt{s}$. Therefore, there is one critical point (a maximum for a binary nucleus and a saddle point for a ternary nucleus) of the formation energy surface F(x,s), which corresponds to the critical nucleus [114]. In order to find the size and composition of the critical nucleus, one should solve the system of partial differential equations ∂F/∂x=0, ∂F/∂s=0 (or, equivalently, $\partial F/\partial N_{AD}=0,\partial F/\partial N_{BD}=0$), which is equivalent to the following:

(13) $$-\frac{\partial \Delta \mu }{\partial x}s+\frac{da}{dx}\sqrt{s}=0,$$

(14) $$-\Delta \mu +\frac{a}{2\sqrt{s}}=0.$$

Here, a is the appropriately normalized surface energy of a ternary III–V nucleus in thermal units [63].

In Gibbs thermodynamics, the surface energy term is independent of the nucleus composition because the surface concentrations of different species should minimize the surface energy. This corresponds to da/dx=0 in the so called Wilemskii approach in the nucleation theory [78,115] (for very small islands consisting of only a few III–V pairs, this approach should be considered approximate). Then, Equations (13) and (14) are reduced as follows:

(15) $$\frac{\partial \Delta \mu }{\partial x}=0$$

in the saddle point, which fully determines the composition of the critical nucleus. Using the Gibbs–Duhem equation, Equation (15) can be further reduced to the equality of the chemical potential differences for AD and BD pairs as follows:

(16) $$\Delta \mu_{AD}=\Delta \mu_{BD}.$$

According to Figure 3a,b, the chemical potentials of AD and BD binaries equal each other only in a narrow range close to one of the corners of the allowed zone of ternary InGaAs compositions. This results in steep nucleation-limited liquid–solid distribution, shown by symbols in Figure 3d.

Presenting the chemical potentials in Equation (16) as functions of the liquid and solid compositions, the nucleation-limited distribution takes the following form:

(17) $$x=\frac{y}{y+\left(1-y\right)e^{-b\left(y\right)-g\left(x,\omega_{s}\right)}}.$$

Here, $g\left(x,\omega_{s}\right)$ is a function of the solid composition and the pseudo-binary interaction parameter. For the majority of III–V materials systems (excluding materials with strongly composition-dependent pseudo-binary interaction parameter, such as InSb_x_As_1−x_), this function is reduced to $g\left(x,\omega_{s}\right)=2\omega_{s}\left(x-1/2\right)$ [78]. This term under the exponent describes the s-shaped behavior of the liquid–solid distribution and the miscibility gaps, similarly to the equilibrium distribution. The function b(y) is rather complex in the general case [78] and depends on the interaction parameters and concentrations of all the elements dissolved in liquid. It has been shown, however, that the b values in most systems are mainly determined by the difference in the chemical potentials for pure elements $\Delta \mu^{0}\equiv \Delta \mu_{AD}^{0}-\Delta \mu_{BD}^{0}=\left(\mu_{A}^{0}-\mu_{AD}^{0}\right)-\left(\mu_{B}^{0}-\mu_{BD}^{0}\right)$ and binary interactions, while the y dependence of b is weak [78].

The nucleation-limited model was used to describe the VLS growth of self-catalyzed [115] and Au-catalyzed [78,114] nanowires and nanowire heterostructures [115,116]. In Ref. [114], the system of equations $\partial F/\partial N_{AD}=0$ and $\partial F/\partial N_{BD}=0$ were solved in the general case beyond the Wilemskii approximation, and compositions of the critical nucleus were calculated as functions of temperature and compositions of the droplet for different ternary III–V systems. In Ref. [115], the nucleation-limited model was further developed and applied to model the compositions of self-catalyzed In_x_Ga_1−x_As, Al_x_Ga_1−x_As, and InAs_x_P_1−x_ nanowires and interfacial profiles in axial heterostructures based on such nanowires. The analytic liquid–solid distribution was obtained in the form given by relationship Equation (17). It has been shown that the liquid–solid distribution of self-catalyzed VLS III–V nanowires is a three-parametric function, which depends on $\left(c_{A}+c_{B}\right)\omega_{AB}$, α, and $\omega_{s}$. It is reduced to a two-parametric function of α and $\omega_{s}$ when $\left|\Delta \mu_{AD}^{0}-\Delta \mu_{BD}^{0}\right|\gg \left|\omega_{AB}\right|$, with $\Delta \mu_{AD}^{0}=\mu_{A}^{0}+\mu_{D}^{0}-\mu_{AD}^{0}$ and $\Delta \mu_{BD}^{0}=\mu_{B}^{0}+\mu_{D}^{0}-\mu_{BD}^{0}$. Finally, in the materials systems with $\omega_{s}≅0$, the liquid–solid distribution is reduced to the Langmuir–McLean equation x=εy/(1+(ε−1)y), with $\varepsilon =e^{\alpha }$. Including a time-dependent material balance equation for y, which includes alternating vapor fluxes of A and B atoms and their sinks due to nanowire growth, enabled analytical descriptions of the interfacial abruptness in axial nanowire heterostructures.

The compositions of Au-catalyzed VLS ternary III–V nanowires in the nucleation-limited regime were studied [78] for In_x_Ga_1−x_As, Al_x_Ga_1−x_As, In_x_Ga_1−x_Sb, and InSb_x_As_1−x_ systems. It has been shown that nanowire composition can vary in a wide range by tuning the liquid composition, with the exceptions including materials systems with high pseudo-binary interaction parameters. In such materials systems that include InGaAs and InSbAs, liquid–solid distributions show miscibility gaps, similar to the previous models. The influence of Au concentration and temperature on liquid–solid distributions has been studied in detail. Ternary and composition-dependent binary interaction parameters in the liquid phase were taken into account. Phase diagrams showing the zones of phase stability or separation were also calculated.

**Figure 4:**
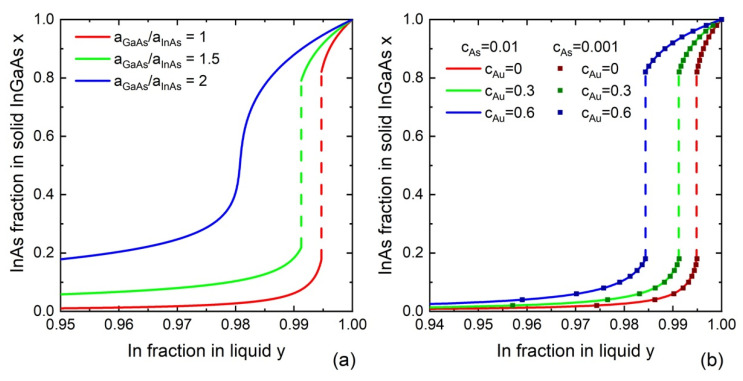

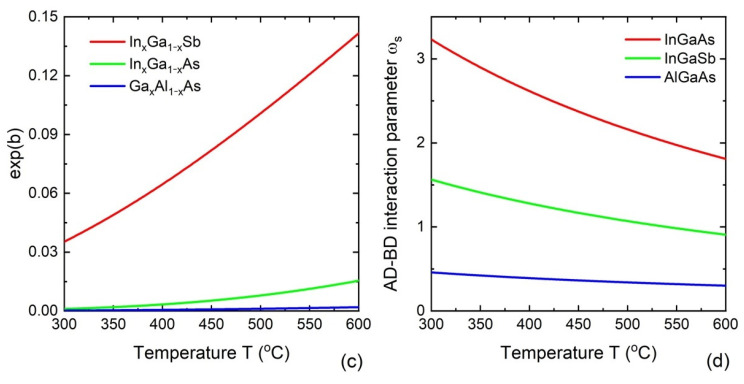
(**a**) Liquid–solid distributions of self-catalyzed In_x_Ga_1−x_As nanowires obtained from Equation (18) at T=450 °C, $c_{Au}=0$, and $c_{As}=0.01$ for different surface energy ratios calculated from the linear Vegard’s law. The dashed curve corresponds to the miscibility gap. (**b**) Liquid–solid distribution of Au-catalyzed In_x_Ga_1−x_As nanowires obtained from Equation (17) for different Au and As concentrations at a fixed T=450 °C. (**c**) $e^{b}$ as a function of temperature for InGaAs, InGaSb, and AlGaAs ternaries at a fixed $c_{As}=0.01$ and $c_{Au}=0$. (**d**) Pseudo-binary interaction parameters $\omega_{InAs-GaAs}$, $\omega_{InSb-GaSb}$, and $\omega_{AlAs-GaAs}$ versus temperature.

When da/dx≠0, the surface energy of the critical island influences the solid compositions and the miscibility gaps. These effects were studied in Ref. [117]. For a composition-dependent a, which is a possible case for ternaries based on group III intermix, Equations (6) and (7) can be reduced as follows:

(18) $$\frac{\Delta \mu_{AD}}{\Delta \mu_{BD}}=1+\frac{\frac{2}{a}\frac{da}{dx}}{1-\frac{2}{a}\frac{da}{dx}x},$$

which is reduced to Equation (16) at da/dx=0. The simplest approximation for composition-dependent surface energy is given by Vegard’s law $a=xa_{AD}+\left(1-x\right)a_{BD}$, which is simply the linear interpolation between the two surface energies of binary liquids, AD and BD. One can also use a convex function of the surface energies of binary liquids, corresponding to Vegard’s law with a bowing parameter α, $a=xa_{AD}+\left(1-x\right)a_{BD}+\alpha x\left(1-x\right)\left(a_{AD}-a_{BD}\right)$. Both models were considered in Ref. [73]. For self-catalyzed VLS nanowires, the $a_{AD}$ and $a_{BD}$ values are reduced to $a_{A}$ and $\;a_{B}$ for pure A and B liquids. Modeling of VLS InGaAs nanowires revealed a small contribution of the composition-dependent surface energy in the case of the center nucleation at the liquid–solid interface away from the triple-phase line where the three phases met for the plausible range of $a_{GaAs}/a_{InAs}$ ratios below 1.5, as demonstrated in Figure 4a. This justifies the use of the Wilemskii approximation. However, further increase in the surface energy ratio results in the complete suppression of the miscibility gap at 450 °C. In the case of nucleation at the triple-phase line, the convex model predicts a much stronger effect than the linear Vegard’s law, with the complete suppression of the miscibility gap already at $a_{GaAs}/a_{InAs}=1.15$.

We now discuss the influence of Au concentration, group V concentration, and temperature on the liquid–solid distributions of ternary III–V nanowires within the Wilemskii approach. Summarizing the effect of Au, it has been found that increasing Au concentration results in an increase in In content in In_x_Ga_1−x_As and In_x_Ga_1−x_Sb nanowires, Ga content in Al_x_Ga_1−x_As nanowires, and As content in InSb_x_As_1−x_ nanowires at a fixed liquid composition y [78]. Figure 4b shows the corresponding results for InGaAs nanowires.

Both chemical potential differences $\Delta \mu_{AD}$ and $\Delta \mu_{AD}$ contain the liquid chemical potential of atom D, $\mu_{D}^{l}$. At da/dx=0, corresponding to $\Delta \mu_{AD}=\Delta \mu_{AD}$, this term vanishes and has no effect on liquid–solid distribution. The concentration of atom D in liquid enters other chemical potentials $\mu_{A}^{l}$ and $\mu_{B}^{l}$ in their binary and ternary interaction terms. However, the concentration of group V atoms in liquid is very low, leading to an almost negligible effect on $\mu_{A}^{l}$ and $\mu_{B}^{l}$. As a result, the influence of the total concentration of group V elements on the liquid–solid distribution is very weak, which is why the solid curves and symbols in Figure 4b are almost identical. Circumventing the uncertainty in the unknown group V concentrations can therefore be considered one of the advantages of the nucleation-limited model.

The temperature effect on nanowire composition is mainly due to its influence on the chemical potential difference between AD and BD pairs (entering the parameter b) and on the pseudo-binary interaction parameter. Temperature dependence of exp(b) for different materials systems is given in Figure 4c. According to these data, b increases with temperature for all systems, which shifts the liquid–solid distribution curves toward x=y. In particular, rising temperature increases the fractions of InAs, InSb, and GaAs in In_x_Ga_1−x_As, In_x_Ga_1−x_Sb, and Ga_x_Al_1−x_As nanowires, respectively. For very low values of exp(b), the distributions become sharp. Within the materials systems considered, the lowest value of the parameter b corresponds to Ga_x_Al_1−x_As ternary.

As mentioned above, the absence or presence of the miscibility gap and its width is entirely determined by the pseudo-binary interaction parameter in thermal units at a given temperature. This parameter decreases with temperature, leading to progressive shrinking and finally the disappearance of the miscibility gap at lower temperatures. Figure 4d shows the temperature dependence of the pseudo-binary interaction parameters in thermal units for InGaAs, InGaSb, and AlGaAs materials systems. Among these systems, interactions between dissimilar III–V pairs are strongest for the InGaAs system with a 7.1% lattice mismatch. This leads to miscibility gaps at typical growth temperatures, which disappear at the critical temperature of T=543 °C, corresponding to $\omega_{InAs-GaAs}=2$. Above this temperature, tuning over the entire compositional range is allowed in thermodynamics and the nucleation-limited model.

To summarize, the nucleation-limited model describes the size and composition of the critical nucleus, which is very small under usual VLS growth conditions (in the order of 10 III–V pairs). Applying this model to fully formed nanowires, whose monolayers consist of at least a thousand III–V pairs, assumes that the solid composition does not change during monolayer growth. The crystal structure of III–V nanowires, which can be either cubic zincblende or hexagonal wurtzite [118], is indeed determined during the nucleation stage and cannot change within one monolayer. This is of course not guaranteed for the composition of ternary III–V islands, and there are no kinetic data on the composition of fractional monolayers due to the low resolution of characterization techniques. Obviously, the nucleation-limited model should work well when the critical nucleus becomes very large, which occurs at low supersaturations (when Δμ→0). This limits the applications of the model and explains why the nucleation-limited composition appears close to the equilibrium composition. When III–V composition is determined in the kinetic growth stage of large (supercritical) island, one should use kinetic models rather than the nucleation-limited model.

### 4.3. Kinetic Models for Liquid–Solid Incorporation

Kinetic models describe the liquid–solid growth of a ternary fractional monolayer of supercritical size. The monolayer is fed by the diffusion fluxes of A, B, and D atoms. The model is based on the material balance equations for the number of AD ($N_{AD}$) and BD ($N_{BD}$) pairs in a ternary ABD island, described by the rate constants of attachment $W^{+}$ and detachment $W^{-}$. In the simplest case, the decoupled binary incorporation rates of AD and BD pairs can be denoted as $dN_{AD}/dt=W_{AD}^{+}-W_{AD}^{-}$ and $dN_{BD}/dt=W_{BD}^{+}-W_{BD}^{-}$. This results in the two kinetic equations in the following forms [119]:

(19) $$\frac{dN_{AD}}{dt}=W_{AD}\left(1-e^{\frac{dF}{dN_{AD}}}\right),$$

(20) $$\frac{dN_{BD}}{dt}=W_{BD}\left(1-e^{\frac{dF}{dN_{BD}}}\right),$$

where F is the formation energy of the island in thermal units. Here, we denote $W_{AD}=W_{AD}^{+}$ and $W_{BD}=W_{BD}^{+}$ for brevity. If the contribution of the surface energy into the formation energy is small, one may use $dF/dN_{AD}=-\Delta \mu_{AD}$ and $dF/dN_{BD}=-\Delta \mu_{BD}$. The binary attachment rates should be proportional to the products of the concentrations of A, D and B, D atoms dissolved in liquid, as follows: $W_{AD}=K_{AD}c_{A}c_{D}$ and $W_{BD}=K_{BD}c_{B}c_{D}$, where $K_{AD}$ and $K_{BD}$ are the crystallization rates which summarize the kinetic growth effects. Then, the steady-state solid composition $x=\left(dN_{AD}/dt\right)/\left(dN_{AD}/dt+dN_{BD}/dt\right)$ becomes the following:

(21) $$x=\frac{1}{1+k\frac{\left(1-y\right)}{y}\frac{\left(1-e^{-\Delta \mu_{BD}}\right)}{\left(1-e^{-\Delta \mu_{AD}}\right)}},$$

where $k=K_{BD}/K_{AD}$. k is a function that depends on the details of material transfer. However, in most studies it was simply put to unity (k=1).

**Figure 5:**
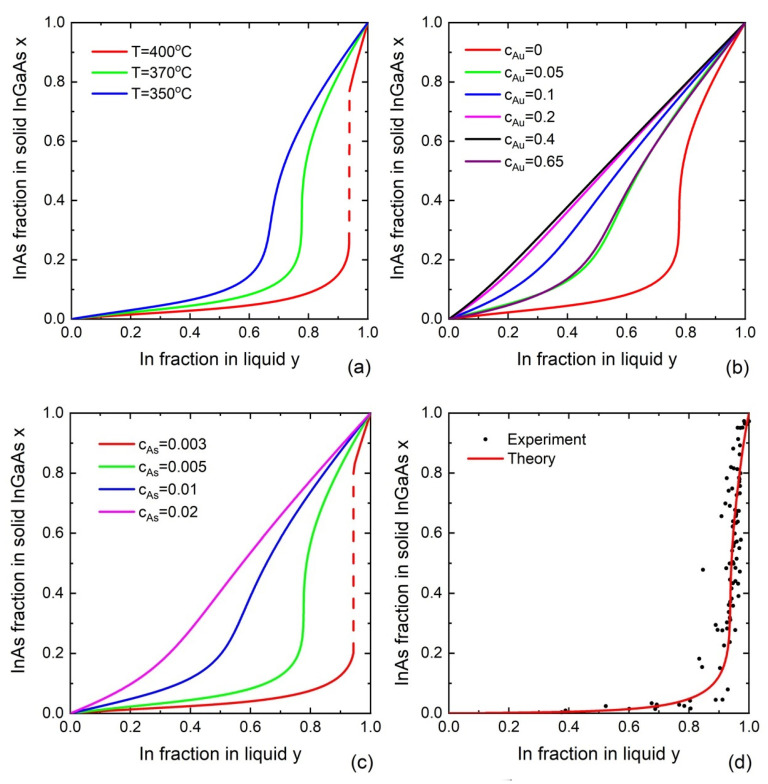
Liquid–solid distributions of In_x_Ga_1−x_As nanowires obtained from Equation (21) for different (**a**) temperatures (at a fixed $c_{As}=0.005$ and $c_{Au}=0$), (**b**) Au concentrations (at a fixed $c_{As}=0.005$ and T=370 °C), and (**c**) As concentrations (at a fixed $c_{Au}=0$ and T=370 °C). The dashed curve corresponds to the miscibility gap. (**d**) Experimental (symbols) and theoretical (line) liquid–solid distribution of Au-catalyzed In_x_Ga_1−x_As nanowires of Ref. [68]. In calculations, we used the values of k=3, T=380 °C, $c_{Au}=0.57$, and $c_{As}=10^{-4}\exp(2.8y^{2})$. The surface energies depend weakly on y so that $a_{InAs}$ increases linearly from 3.53 to 3.66 and $a_{GaAs}$ from 4.05 to 4.16 as y increases from 0 to 1.

Figure 5a shows the InAs fraction in self-catalyzed In_x_Ga_1−x_As nanowires as a function of the liquid composition y, calculated using Equation (21) for different temperatures of T=350, 370, and 400 °C. In contrast to the nucleation-limited regime, the InAs fraction decreases with temperature. It should be mentioned, however, that we used a constant value of $c_{As}=0.005$ in all calculations, while the actual As concentration is expected to decrease for higher temperatures at a given As flux. To our knowledge, no comprehensive investigation of the temperature effect on liquid–solid distributions has been published so far.

We now analyze the influence of Au concentration on the liquid–solid distribution of self-catalyzed In_x_Ga_1−x_As nanowires. Figure 5b shows the non-monotonic behavior of this dependence. Increasing the Au concentration from 0 (the self-catalyzed VLS growth) to 0.2 changes the distribution from the Γ shape at $c_{Au}=0$ to linear at $c_{Au}=0.2$. The growth regimes with a low Au concentration in the range from 0.05 to 0.2 correspond to the Au-catalyzed VLS growth with group-III-rich droplets. Such regimes can be achieved under low V/III flux ratios due to droplet enrichment with the arriving group III species [18]. In the intermediate range of Au concentrations ($c_{Au}=0.2-0.5$), it has almost no effect on the curves. A further increase in $c_{Au}$ retains the Γ shape of the distribution in such a way that the curves at $c_{Au}=0.05$ and $c_{Au}=0.65$ are almost identical.

The liquid–solid distributions of self-catalyzed In_x_Ga_1−x_As nanowires at different As concentrations are given in Figure 5c. It is seen that $c_{As}$ has a very significantly influence on the shapes of liquid–solid distributions. This feature is different from the nucleation-limited model, where $c_{As}$ has almost no effect on solid composition. The liquid–solid distribution obtained from the kinetic model is similar to the nucleation-limited curve, only at low As concentrations where it predicts the miscibility gap. At higher As concentrations, the curves become more and more linear and finally approaches x=y. The miscibility gap shrinks with increasing As concentration. We note, however, that the As concentration remains a parameter in the kinetic model with decoupled binary fluxes shown in Equations (19) and (20). We suspect that this model is valid only in D-rich growth regimes (an As-rich regime in this example), where the growth kinetics of a ternary island is limited by the material transport of A and B atoms (In and Ga in this example). At very small As concentrations, or more generally, concentrations of atom D, the situation is reversed and the growth may become D-limited and enriched in A and B species, which should have an effect on the liquid–solid distribution. This important question requires additional study which is presented elsewhere.

The kinetically limited composition of ternary III–V nanowires in the diffusion-induced growth regime was studied in [120]. The attachment rates of the AD and BD pairs were presented in terms of diffusive fluxes, namely $W_{AD}=ChJ_{A}J_{D}/\left(J_{A}+J_{D}\right)$ and $W_{BD}=ChJ_{B}J_{D}/\left(J_{B}+J_{D}\right)$. Here, C is the perimeter, h is the width of an island, and $J_{i}$ represents the diffusion fluxes of A, B, and D atoms. The obtained liquid–solid distribution is in agreement with the Stauffer result [119]. Under the assumption of $J_{D}\ll J_{A}$ and $J_{D}\ll J_{B}$, one has $W_{AD}=W_{BD}$. This simplifies Equations (19) and (20) as follows:

(22) $$x=\frac{1}{1+\frac{\left(1-e^{-\Delta \mu_{BD}}\right)}{\left(1-e^{-\Delta \mu_{AD}}\right)}}.$$

This equation was solved numerically for different Au concentrations and temperatures. This enabled an analysis of compositions in In_x_Ga_1−x_As nanowires and their stability. The model provided an explanation for the kinetic suppression of the miscibility gap. The compositional tuning around x=0.5 requires that the 𝑦 values are very close to one, which corresponds to In-rich droplets.

The effects of growth temperature, group V concentration, and Au concentration on the liquid–solid distributions of III–V ternary nanowires was studied in detail in Ref. [121] based on Equation (21). The evolution of the liquid–solid distributions of In_x_Ga_1−x_As nanowires from the nucleated-limited to the kinetically controlled shape was also investigated. In particular, the composition of the supercritical island was obtained in the form of a linear combination of the nucleation-limited kinetic shapes. The solid composition is determined by the ratio of the critical size to the island size. Considering the stationary case, the liquid–vapor distribution was obtained in the following form:

(23) $$\frac{1-y}{y}=\frac{1-z}{z}\gamma$$

where $\gamma =\chi_{B}\tau_{B}/\chi_{A}\tau_{A}$ in the case of IIIV_x_V_1−x_ ternaries, $\gamma =\left(R\chi_{B}+2\varphi_{B}\lambda_{B}\right)/\left(R\chi_{A}+2\varphi_{A}\lambda_{A}\right)$ in the case of III_x_III_1−x_V ternaries, and $\tau_{i}$ represents the characteristic desorption times from liquid. This enabled the linking of the compositions of the nanowire and vapor

(24) $$x=\frac{1}{1+k\gamma \frac{1-z}{z}\frac{\left(1-e^{-\Delta \mu_{BD}}\right)}{\left(1-e^{-\Delta \mu_{AD}}\right)}}.$$

The calculated vapor–solid distribution of In_x_Ga_1−x_Sb nanowires is in agreement with the experimental data [80,122].

The correlation between the compositions of the liquid droplet and VLS III–V ternary nanowires was obtained for the first time in Ref. [68]. The Au-catalyzed VLS growth of In_x_Ga_1−x_As nanowires was studied in situ using ETEM. The growth conditions employed in this ETEM study were specific, with no substrate and too-low vapor pressure compared to the typical MOVPE conditions. Nevertheless, the in situ technique provided unique information about the nanowire growth process. In_x_Ga_1−x_As nanowires were grown at a temperature of 380 °C and an average Au concentration $c_{Au}\approx 0.6$. In situ data demonstrated that (i) the InGaAs composition can vary over the entire range, with no miscibility gap present in nanowires; and (ii) the synthesis of nanowires with a significant InAs content (x>0.1) requires very high In concentrations in the droplet ($c_{In}/\left(c_{In}+c_{Ga}\right)≅0.9$). Theoretical calculations based on Equations (19) and (20) showed good agreement with the compositional data, as demonstrated in Figure 5d [68]. The data were obtained using in situ nanowire growth monitoring in ETEM. InGaAs nanowires were grown by the Au-catalyzed MOVPE at 380 °C, using 30 nm diameter colloidal Au nanoparticles under a gas phase V/III ratio of 1000 and variable fluxes of TMIn and TMGa.

Homogenous In_x_Ga_1−x_N nanowires with a controlled In composition of up to 90% were grown on GaN/c-Al_2_O_3_ templates by catalyst-free HVPE [123]. In Ref. [124], HVPE growth and the composition of In_x_Ga_1−x_N nanowires were investigated as a function of temperature. Special emphasis was put on the kinetic suppression of the miscibility gap in this material system. Using lattice gas approximation [125], the liquid–solid distribution was obtained in the form similar to that in Equation (21):

(25) $$x=\frac{1}{1+ke^{\mu_{B}^{L}-\mu_{A}^{L}}\frac{\left(1-e^{-\Delta \mu_{BD}}\right)}{\left(1-e^{-\Delta \mu_{AD}}\right)}}.$$

The only difference between Equations (21) and (25) is the coefficient before the exponential terms, which can be accounted for by the modification of k (which remains generally unknown in all models with decoupled binary fluxes). Using the relationship between the liquid composition and the indium fraction in vapor and using some additional assumptions, the vapor–solid distribution was obtained in the following form:

(26) $$z=\frac{1}{1+\gamma \left(\frac{1-x}{x}+\delta \left(1-x\right)e^{\omega_{s}x^{2}}\right)}.$$

Here, γ describes the difference in In and Ga adatom diffusivities and δ depends on the vapor supersaturation with respect to solids. In purely kinetic growth regimes (δ→0), Equation (26) is simplified to the one-parametric Langmuir–McLean equation and the miscibility gap disappears. The developed model is capable of describing the compositions within the thermodynamic miscibility gap of InGaN alloys.

An important limiting case of the kinetic model occurs at high supersaturations of both AD and BD pairs, corresponding to $\Delta \mu_{AD}\gg 0$ and $\Delta \mu_{BD}\gg 0$, respectively. The incorporation rates in Equations (19) and (20) then simply equal the attachment rates. The solid composition takes the following form:

(27) $$x=\frac{1}{1+k\frac{1-y}{y}},$$

which is similar to Equation (26) at δ→0. However, it applies to the liquid–solid distribution x(y), while Equation (26) applies to the vapor–solid distribution x(z). The equivalent formula for the liquid–solid growth is given as follows:

(28) $$x=\frac{K_{AD}c_{A}}{K_{AD}c_{A}+K_{BD}c_{B}}.$$

In the purely kinetic limit, the nanowire composition is simply given by the ratio of the growth rate of the AD binary, $K_{AD}c_{A}c_{D}$, over the total growth rate, $c_{D}\left(K_{AD}c_{A}+K_{BD}c_{B}\right)$, where the unknown $c_{D}$ is cancelled in the ratio. In most works [126,127,128], this scenario is not considered by itself, but is coupled with the materials’ balance in the droplet, which can be related to the vapor composition z.

To sum up, the kinetic models for liquid–solid incorporation in their present state are quite sensitive to growth parameters such as temperature, Au concentrations, and D concentrations. This gives flexibility for fitting very different liquid–solid distributions, but some parameters remain unknown. An obvious disadvantage of the model with decoupled fluxes is the unknown concentration of atom D, which serves as an external parameter of the model. However, the clear advantage is that the model describes the kinetic suppression of miscibility gaps at high supersaturations. This effect is seen experimentally in most InGaAs and InGaN nanowires but cannot be described within the equilibrium or nucleation-limited models.

## 5. Vapor–Solid Incorporation Models

In this section, we consider the models that focus on the description of vapor–solid compositions of III–V ternary layers, VS nanostructures and nanowires, and, in some cases, VLS nanowires. These approaches can be divided into reaction-limited, transport-limited, empirical, and kinetic models.

### 5.1. Reaction-Limited Models

The growth model of Ref. [129], which describes the influence of the III/V flux ratio on the solid composition and is valid at any III/V flux ratio, considers chemical reactions at the interface as follows:

(29) $$\frac{1}{m}A_{m}\left(g\right)+\frac{1}{l}D_{l}\left(g\right)=AD\left(s\right),$$

(30) $$\frac{1}{m}B_{m}\left(g\right)+\frac{1}{l}D_{l}\left(g\right)=BD\left(s\right),$$

with the equilibrium constants

(31) $$K_{AD}=\frac{a_{AD}}{p_{D_{l}}^{1/l}p_{A_{m}}^{1/m,}},$$

(32) $$K_{BD}=\frac{a_{BD}}{p_{D_{l}}^{1/l}p_{B_{m}}^{1/m}}$$

for the corresponding reactions and partial pressures, i.e., $p_{A_{m}}$, $p_{B_{m}}$, and $p_{D_{l}}$. Here, m and l are the numbers of atoms in a precursor molecule participating in the reaction (for example, one for AsH_3_ and four for As_4_ vapors, and one for GaCl and Ga(CH_3_)_3_). Using the regular solution model, the activities of the AD and BD pairs, $a_{AD}$ and $a_{BD}$, respectively, are given as follows:

(33) $$a_{AD}=xe^{\omega_{s}{\left(1-x\right)}^{2}},$$

(34) $$a_{BD}=\left(1-x\right)e^{\omega_{s}x^{2}}.$$

The solid composition is given as follows:

(35) $$x=\frac{n_{A_{m}}^{0}-n_{A_{m}}}{n_{A_{m}}^{0}-n_{A_{m}}+n_{B_{m}}^{0}-n_{B_{m}}},$$

where $n_{A_{m}}^{0}$ and $n_{B_{m}}^{0}$ are the initial number of moles of the corresponding precursor molecules in the vapor phase, and $n_{A_{m}}$ and $n_{B_{m}}$ are the equilibrium number of moles of $A_{m}$ and $B_{m}$ in the vapor phase, respectively.

Taking into account the stoichiometry, we obtain:

(36) $$l\left(n_{D_{l}}^{0}-n_{D_{l}}\right)=m\left(n_{A_{m}}^{0}-n_{A_{m}}\right)+m\left(n_{B_{m}}^{0}-n_{B_{m}}\right).$$

Using the perfect gas equation of state and Equations (31)–(36), one can solve this system for the four unknows, namely x, $p_{A_{m}}$, $p_{B_{m}}$, and $p_{D_{l}}$, for a given temperature and the starting molar flow rates of the precursors. The model can also be applied for modeling the vapor–solid distributions in binary IV_x_IV_1−x_ materials systems, for example Si_x_Ge_1−x_ layers [130,131]. A similar approach has been used to describe the incorporation of As and P into GaInPAs during MBE growth [132].

In some models, the ratio of $p_{A_{m}}$ to $p_{B_{m}}$ pressure at the growing surface is taken proportional to the same ratio in the source vapor [133] as follows:

(37) $$\frac{z}{1-z}=\frac{p_{A_{m}}^{0}}{p_{B_{m}}^{0}}\approx \frac{p_{A_{m}}}{p_{B_{m}}}.$$

The vapor–solid distribution can then be obtained in the following form [133,134]:

(38) $$z=\frac{1}{1+{\left[\frac{K_{AD}}{K_{BD}}\frac{1-x}{x}e^{\omega_{s}\left(2x-1\right)}\right]}^{m}},$$

which is similar to the equilibrium distribution for a precursor with m atoms A and B. The disadvantage of this assumption is the lost effect of the III/V flux ratio on the composition, which is present in the initial model. Equation (38) was used to model the compositions of In_x_Ga_1−x_As layers grown by VPE in an (In-Ga)-AsCl_3_-As-H_2_ system with an In-Ga metal source, which produced chloride precursors InCl and GaCl and As_4_ vapor in carrier gas H_2_ [134].

**Figure 6:**
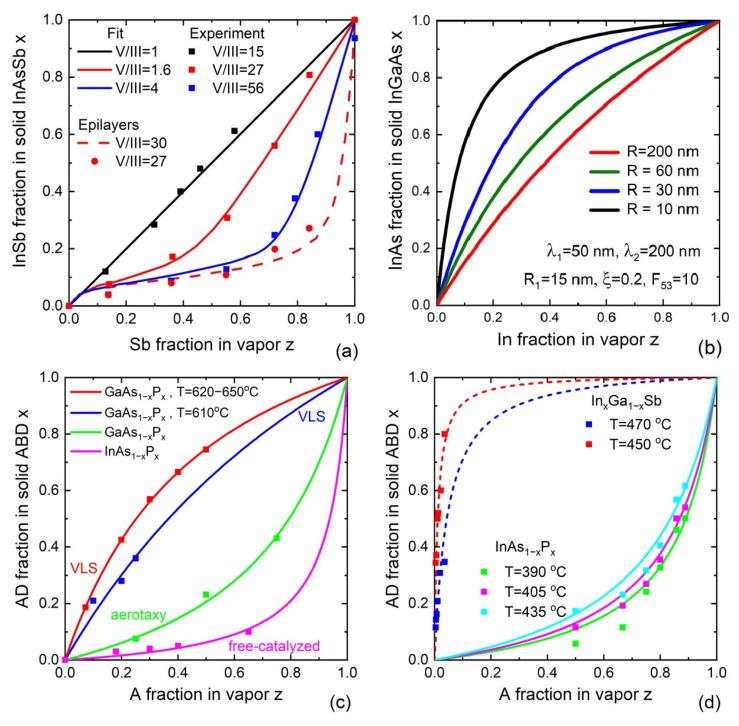
(**a**) Experimental vapor–solid distributions of InAs_1−x_Sb_x_ nanowires under different V/III ratios (squares) and their fits by the reaction-limited model (solid lines) compared to the measured distribution in epilayers (circles) and their fits (dashed line) by the same model [44]. The fitting values of V/III flux ratios for nanowires are much lower than V/III flux ratios in vapor in all cases. (**b**) Theoretical vapor–solid distributions of Au-catalyzed In_x_Ga_1−x_As nanowires of Ref. [126], calculated for different nanowire radii within the transport-limited model. (**c**) Experimental (symbols) and theoretical (lines) vapor–solid distributions of GaAs_1−x_P_x_ and InAs_1−x_P_x_ nanowires compiled from different works [137,138,139,140]. The fits were obtained using the empirical model [137,138,139,140]. (**d**) Experimental (symbols) and theoretical (lines) vapor–solid distributions for InAs_1−x_P_x_ and In_x_Ga_1−x_Sb nanowires grown at different temperatures, compiled from different works [122,141]. The fits were obtained from the empirical model [141] (solid lines) and from Equation (46) (dashed lines) at T=470 °C, $K_{B/A}=0.0453$ and T=450 °C, $K_{B/A}=0.0113$.

In the field of III–V ternary nanowires, the reaction-limited model was used to describe the compositions of Au-catalyzed MOVPE [44] and catalyst-free selective area MOVPE [135] and MBE [136] InAs_1−x_Sb_x_ nanowires. It was shown that a decrease in V/III ratio results in a significant enhancement in the amount of Sb incorporated into the nanowires [44], as shown in Figure 6a. The amount of Sb incorporated into the nanowires appeared much higher compared to that of the epilayers [44]. The reaction-limited model requires reliable data on the equilibrium constants of different reactions in a given epitaxy technique. This approach does not take into account the surface diffusion of group III adatoms. This explains why the values of the V/III ratio used to fit the experimental data of Ref. [32] were different from the actual V/III ratios in vapor. Material fluxes of group III atoms into the droplets were enhanced by surface diffusion from the nanowire sidewalls, which led to a much lower actual V/III ratio for the fluxes entering the nanowires compared to the values set by the vapor fluxes.

### 5.2. Transport-Limited Models

The VLS growth models of this type are based on material balance equations for the number of atoms (or concentrations) in the liquid droplet, which are influenced by the incoming vapor fluxes and the desorption fluxes leaving the droplet. This, in principle, allows for the simultaneous determination of the steady-state liquid–solid and vapor–solid distributions. The solid composition is given by the ratio of the growth rate of the AD binary at the interface over the total VLS growth rate, as in Equation (28). In the simplest case without any rejected fluxes leaving a ternary island, material balance in the droplet is given by:

(39) $$K_{AD}c_{A}c_{D}=V_{A}-U_{A}c_{A}^{\eta },$$

(40) $$K_{BD}c_{B}c_{D}=V_{B}-U_{B}c_{B}^{\eta },$$

(41) $$\frac{dL}{dt}=c_{D}\left(K_{AD}c_{A}+K_{BD}c_{B}\right)=V_{D}-U_{D}c_{D}^{\kappa },$$

Here, $V_{i}$ represents the atomic vapor fluxes; $U_{i}$ represents the kinetic coefficients that determine the desorption fluxes for each type of atom (i=A,B and D); and dL/dt represents the nanowire growth rate. The power exponents η and κ account for the fact that group V atoms usually desorb in the form of dimers, in which case η or κ equals 2. These equations can be linearized assuming a small variation of the group V concentrations in the droplet. The exact solution for vapor–solid distribution following from Equations (39)–(41) at η=κ=1 is given as follows [126]:

(42) $$x=\frac{z}{z+f\left(x\right)\gamma \left(1-z\right)}.$$

Here, f(x) is a complex function of the solid composition given in Ref. [126]. It has been shown that the solid composition in nanowires differs from the vapor composition due to (i) different diffusion fluxes of A and B adatoms of group III elements, determined by their effective diffusion lengths, and (ii) asymmetry of the sinks due to desorption or backward diffusion from the droplet onto the nanowire sidewalls. The typical vapor–solid distribution following from Equation (42) is shown in Figure 6b. The radius dependence is due to the surface diffusion of Ga and In atoms on the sidewalls of InGaAs nanowires. Generally, there is a significant enhancement in the incorporation of element A for thinner nanowires if its diffusion length is larger compared to element B. The incorporation of such an element also increases with the V/III flux ratio.

There are three important limiting cases of Equation (42). First, for III_x_III_1−x_V nanowires, the desorption fluxes are often negligible, corresponding to $U_{A}=U_{B}=0$. This yields a simple vapor–solid distribution of the Langmuir–McLean type as follows:

(43) $$x=Z=\frac{z}{z+\gamma \left(1-z\right)},$$

where γ is given by Equation (5) for short nanowires and Equation (6) for long nanowires. This expression was used to model the spontaneous Au-catalyzed Al_x_Ga_1−x_As core-shell nanowire heterostructures grown by MBE [127]. This situation was also considered in Ref. [142]. The second limiting case is the self-catalyzed VLS growth of III–V_x_V_1−x_ nanowires. At $c_{D}\approx 1$, Equation (42) takes the form of Equation (43) [128] with γ given by

(44) $$\gamma =\frac{1+U_{A}/K_{AD}}{1+U_{B}/K_{BD}}.$$

In Au-catalyzed VLS growth of III–V_x_V_1−x_ nanowires, γ changes to the following:

(45) $$\gamma =\frac{1-c_{U}+U_{A}/K_{AD}}{1-c_{U}+U_{B}/K_{BD}}.$$

The third limiting case occurs when the solid composition equals the liquid composition (x=y), which corresponds to the rare case of $K_{AD}=K_{BD}$. This situation was considered in Ref. [46].

There are some general advantages of transport-limited models. First, they provide, explicitly or implicitly, the vapor–solid distribution, which is more relevant for experimentalists because it is controlled by vapor fluxes that can be measured very accurately. The liquid phase, which is always present in VLS nanowires and whose composition is usually unknown, plays the role of an interface between the liquid and solid phases rather than the mother phase for the nucleation and growth of ternary nanowire monolayers. Second, these models provide axial and radial growth rates of ternary nanowires and hence can be used to control the nanowire morphology. On the other hand, modeling requires some external parameters, such as the diffusion lengths of group III adatoms on different surfaces and the evaporation rates of group V atoms from liquid, which may depend on the droplet composition.

### 5.3. Empirical Models

One of the simplest methods to describe the chemical composition of III–V ternary layers and different ternary nanostructures, including nanowires, is the one-parametric growth model. It is assumed that the solid composition is determined by the B-to-A incorporation ratio, $K_{B/A}$. In this case, the vapor–solid distribution is again reduced to the Langmuir–Mclean formula:

(46) $$x=\frac{z}{z+K_{B/A}\left(1-z\right)}.$$

For group III adatoms, $K_{B/A}$ may include the parameters γ related to different diffusion lengths of A and B adatoms.

Initially, this model was applied to 2D growth of GaAs_1−x_P_x_ [143] and InAs_x_P_1−x_ [144] epilayers. It was capable of describing the non-linear shapes and the temperature dependence of the measured vapor–solid distributions. The temperature dependence of $K_{B/A}$ was found to follow Arrhenius behavior, where the logarithm of the fitting values of $K_{B/A}$ scales linearly with 1/T within a temperature range employed in the growth experiments [143]. Therefore, its temperature is often taken in the form $K_{B/A}\sim e^{-E_{A}/k_{B}T}$, where $E_{A}$ is an activation energy extracted from the fits. However, Arrhenius-type behavior does not hold in a wider temperature range [122].

This one-parametric equation has been used to understand the compositions of VLS and catalyst-free VS III–V_x_V_1−x_ and III_x_III_1−x_-V nanowires in different materials systems. Figure 6c summarizes the published data on the nanowire compositions. In Ref. [138], self-catalyzed GaAs_1−x_P_x_ nanowires were grown by MBE at different temperatures, ranging from 620 °C to 650 °C. The enhanced incorporation of P atoms into nanowires relative to 2D layers was demonstrated. In contrast to group-V-limited VLS growth of nanowires, planar growth always proceeds under group-V-rich conditions. In this case, the incorporation rate of P atoms is much slower compared to As atoms, because P is more volatile than As [145]. This may also explain low P incorporation rates observed in catalyst-free InAs_1−x_P_x_ nanowires grown by MBE in the temperature range of 470 °C to 490 °C [139]. In Ref. [137], MBE growth of self-catalyzed GaAs_1−x_P_x_ nanowires was performed at a high temperature of 610 °C. The authors mentioned the spontaneous formation of core–shell structures with different compositions in the cores and shells, which may cause inaccuracies in the measured compositions. In any case, the incorporation efficiencies of P atoms were slightly lower than in Ref. [138]. Au-catalyzed GaAs_1−x_P_x_ nanowires with GaP fractions from up to 0.43 were also synthesized by the substrate-free aerotaxy at 550 °C [140]. These data are summarized in Figure 6c.

The temperature behaviors of the vapor–solid distributions for InAs_1−x_P_x_ and In_x_Ga_1−x_Sb nanowires are summarized in Figure 6d. In Ref. [141] Au-catalyzed InAs_1−x_P_x_ nanowires were grown by chemical beam epitaxy at different temperatures ranging from 390 °C to 435 °C. As for III_x_III_1−x_-V nanowires, a systematic study of In_x_Ga_1−x_Sb nanowire growth and composition was published in Ref. [122]. Importantly, the growth process saturated at high temperatures above 510 °C. The use of Arrhenius temperature dependence for $K_{B/A}$ did not work at high temperatures. In both cases considered, the vapor–solid distributions became more linear and approached x=z in the high-temperature range.

Most of the considered models can be reduced to Equation (46) in the limiting cases. First, the nucleation model given by Equation (17) at $\omega_{s}=0$, together with Equation (23), yields Equation (46). In this case, $K_{B/A}=\gamma \exp\left(-b\right)$. Second, a similar expression can be obtained from the equilibrium model at $\omega_{s}=0$ because its main result has exactly the same form as Equation (17). Third, the transport-limited model given by Equation (42) with $U_{A}K_{AD}=U_{B}K_{BD}$ yields f(x)=1, which reduces the vapor–solid distribution to Equation (46) with $K_{B/A}=\gamma$. For example, this result is valid for ternaries based on group III intermix at low desorption rates of A and B atoms ($U_{A}=U_{B}=0$).

It is worth mentioning that one of the first models for vapor–solid distribution simply assumed that the incorporation rate of A atoms is linear in the vapor composition [146]. This yields the simplest possible formula as follows:

(47) $$x=K_{A}z,$$

with a temperature-dependent $K_{A}$. Clearly, this model can work only for very low x and z, which is more relevant for doping than for ternary materials whose composition varies over a wide range.

### 5.4. Kinetic Model for Vapor–Solid Growth

Perhaps the most general form of vapor–solid distribution has recently been obtained in Ref. [147]. The diffusion-induced growth process of III–V ternary materials and nanomaterials was considered in different geometries, including planar layers, nanomembranes, and horizontal and vertical nanowires grown by selective area epitaxy. Under certain assumptions, the obtained vapor–solid distribution can also be applied to VLS nanowires with a catalyst droplet on top. The form of the vapor–solid distribution was shown to remain identical for a wide range of geometries, while the coefficients entering the equation contained thermodynamic factors, kinetic constants of the material transport, and geometrical parameters of the structure. This vapor–solid distribution pertains for D-rich growth of an A_x_B_1−x_D ternary material, which is controlled by the diffusion fluxes of A and B adatoms, and should be applicable in a wide range of ternary nanostructures based on group III intermix. The diffusion fluxes of AD and BD pairs into the solid phase were obtained in the general form as follows:

(48) $$j_{A}=\;\Lambda_{A}\left(I_{A}-\frac{n_{A}^{*}\left(x\right)}{T_{A}}\right),j_{B}=\;\Lambda_{B}\left(I_{B}-\frac{n_{B}^{*}\left(x\right)}{T_{B}}\right).$$

Here, $\Lambda_{k}$ represents the effective diffusion lengths of A and B adatoms, which depend on a particular geometry (separation P between the steps or islands, width and height of a nanomembrane, length and radius of planar or vertical nanowire), and $T_{k}$ represents the effective lifetimes of A and B adatoms on a surface, which may also depend on geometry. The x-dependent functions $n_{A}^{*}\left(x\right)$ and $n_{B}^{*}\left(x\right)$ are given by the following:

(49) $$n_{A}^{*}=\frac{n_{A}^{eq}n_{D}^{eq}}{n_{D}}xe^{\omega_{S}{\left(1-x\right)}^{2}},n_{B}^{*}=\frac{n_{B}^{eq}n_{D}^{eq}}{n_{D}}\left(1-x\right)e^{\omega x^{2}},$$

which describe the “repulsion” of A and B adatoms from the growing interface. The quantities $n_{A}^{eq}n_{D}^{eq}$ and $n_{B}^{eq}n_{D}^{eq}$ are proportional to the temperature-dependent activities of binary AD and BD solids, whereas $n_{D}$ is a spatially uniform surface concentration of adatom D under D-rich conditions.

Whenever the diffusion fluxes are given by Equation (48), the vapor–solid distribution has the following form:

(50) $$z=\frac{x}{c+\left(1-c\right)x}\left[1+\left(1-x\right)\left(cAe^{\omega_{S}{\left(1-x\right)}^{2}}-Be^{\omega_{S}x^{2}}\right)\right],$$

with coefficients

(51) $$c=\frac{\Lambda_{A}}{\Lambda_{B}},A=\frac{n_{A}^{eq}n_{D}^{eq}}{(I_{A}+I_{B})T_{A}n_{D}},B=\frac{n_{B}^{eq}n_{D}^{eq}}{(I_{A}+I_{B})T_{B}n_{D}}.$$

The form of this distribution is similar to that of Equation (21). However, it relates the solid composition to the composition of vapor rather than a liquid droplet in VLS nanowires. In the limiting cases, this kinetic model is reduced to the equilibrium model (at zero fluxes $j_{A}\;$ and $j_{B}$) or to the Langmuir–McLean formula (at A→0 and B→0).

**Figure 7:**
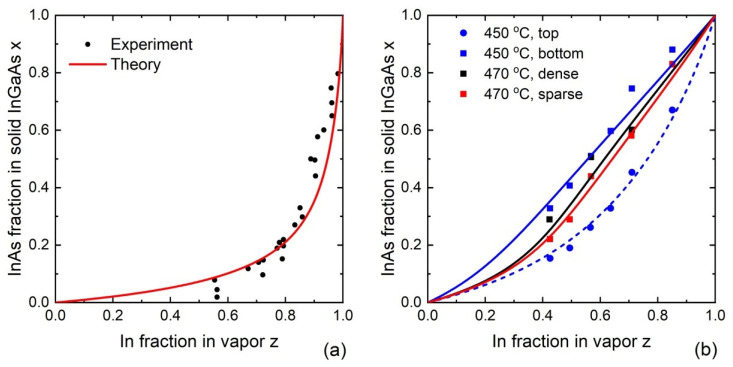
(**a**) Vapor–solid distribution of 2D InGaAs layers of Ref. [134] (symbols), fitted by Equation (50) with the parameters given in Table 1 (solid line) [147]. (**b**) Vapor–solid distribution of Au-catalyzed VLS InGaAs nanowires of Ref. [16] (symbols), fitted by Equation (50) with the parameters given in Table 1 [147].

Figure 7 shows a comparison of the vapor–solid distributions for 2D In_x_Ga_1−x_As layers (Figure 7a) and Au-catalyzed VLS nanowires (Figure 7b). The fitting parameters are summarized in Table 1. In_x_Ga_1−x_As layers of Ref. [134] were grown by VPE in an (In-Ga)-AsCl_3_-As-H_2_ system with an In-Ga metal source producing the chloride precursors InCl and GaCl and As_4_ vapor in the carrier gas H_2_, at a temperature of 750 °C. Au-catalyzed InGaAs nanowires of Ref. [16] were grown by MOVPE using 50 nm diameter Au aerosol nanoparticles deposited onto InAs(111)B substrates, at two different temperatures of 450 °C and 470 °C. Different vapor compositions were achieved by changing the fluxes of TMIn and TMGa precursors. For 3 μm long nanowires grown at 450 °C with a V/III flux ratio of 12.6, composition was measured at the top and bottom. At a temperature of 470 °C and a V/III flux ratio of 6.32, 1.2 μm long InGaAs nanowires were grown with two different surface densities of Au nanoparticles, corresponding to the average distance between the nanowires of 316 nm (dense nanowires in Table 1) and 707 nm (sparse nanowires in Table 1). For these nanowires, the composition was measured at the top. Overall, Figure 7 demonstrates that Au-catalyzed VLS growth allows one to achieve smoother vapor–solid distribution compared to 2D layers. The difference is most likely related to a high growth temperature of 750 °C employed for the growth of 2D layers, which is closer to equilibrium and leads to non-linearity of the curve. No miscibility gap was present in VLS InGaAs nanowires at 450 °C, which shows again that it can be suppressed by fast growth kinetics far from equilibrium conditions.

## 6. Model Comparison for VLS Ternary Nanowires

Analysis of the VLS growth models describing the liquid–solid incorporation and the liquid–solid distribution allows one to draw some general conclusions regarding the qualitative effects of temperature and concentrations of D and U elements on the composition of III–V ternary nanowires. These conclusions are summarized in Table 2, where AD corresponds to an element for which $\Delta \mu_{AD}^{0}<\Delta \mu_{BD}^{0}$.

Table 3 summarizes the reviewed models for nanowire composition, the governing expressions, advantages, and drawbacks, which are described in detail in this review.

## 7. Conclusions

Analysis of different models for compositions of III–V ternary nanowires grown by different epitaxy techniques via VLS or VS mechanisms shows that the general form of theoretical liquid–solid and vapor–solid distributions depends primarily on the modeling approach (nucleation-limited or kinetically controlled growth) and much less on the growth method. However, the growth conditions employed in different epitaxy techniques (MBE, MOVPE, or HVPE) are different, and these differences should be carefully accounted for when selecting the appropriate modeling approach for each technique. Within a given approach, the liquid–solid and vapor–solid distributions are similar. The coefficients entering the governing equations are different and may depend on the liquid composition for VLS nanowires or the diffusion lengths of group III adatoms, III/V flux ratio, pitch of the array, and other details of the nanowire ensemble and the substrate surface for VS nanowires. These factors may largely affect the resulting nanowire compositions. More efforts should be put on studying the vapor–solid distributions and checking the corresponding growth models, because such measurements are easily accessible. The remaining uncertainty in the parameters of the liquid–solid incorporation models, such as the unknown concentration of different elements in the droplet, is inacceptable but may be circumvented using in situ ETEM monitoring [68]. However, the absence of the substrate and too-low vapor pressure compared to MOVPE bring some limitations for the application of such data. Existing compositional models should be developed to take into account monolayer propagation dynamics and nucleation probabilities [149,150,151]. The complex temperature dependence of the compositional trends in VLS and VS nanowires is not fully understood so far and requires further studies.

In our opinion, existing theoretical approaches to modeling the compositions of III–V A_x_B_1−x_D nanowires should be extended beyond the approximation of D-rich growth (such as group-V-rich growth for ternaries based on group III intermix), which allow one to access the V/III flux ratio dependence of the observed compositions and may bring new features to the compositional control in III–V ternary nanowires in general. To this end, the seminal work of Biefeld [129] remains the only approach to access the behavior of vapor–solid distributions under a varying V/III flux ratio, but this model is restricted to the reaction-limited growth kinetics of epilayers and is not adopted for nanowires. Time-dependent generalizations of compositional models, which are used to model the interfacial abruptness in III–V nanowire heterostructures [21,111,115,116,127,148], were beyond the scope of this work (a review can be found, for example, in Ref. [142]). However, they are absolutely required for tuning the properties of device-oriented nanowire structures and should rely upon reliable models for steady-state liquid–solid or vapor–solid distributions.

## Figures and Tables

**Table 1:** Parameters of III–V ternary systems.

Ref.	System	T (°C)	ω	A	B	c
[134]	Planar In_x_Ga_1−x_As layers	750	1.4119	0.7	0	0.12
[16]	Dense Au-catalyzed VLS In_x_Ga_1−x_As nanowires on InAs(111)B	470	2.2846	0.25	0	0.98
[16]	Sparse Au-catalyzed VLS In_x_Ga_1−x_As nanowires on InAs(111)B	470	2.2846	0.25	0	0.85
[16]	Tops of Au-catalyzed VLS In_x_Ga_1−x_As nanowires on InAs(111)B	450	2.3728	0.1	0	0.95
[16]	Bottoms of Au-catalyzed VLS In_x_Ga_1−x_As nanowires on InAs(111)B	450	2.3728	0.1	0	0.35

**Table 2:** The effect of the parameters on liquid–solid composition dependence within different models.

Models	Equilibrium and Nucleation-Limited	Kinetically Controlled ^2^
Temperature effect	AD content increases	BD content increases
Catalyst concentration effect	AD content increases ^1^	AD content increases at low $c_{U}$ decreases at high $c_{U}$
Effect of group V total concentration	Almost no effect	AD content increases

^1^ Applied to In_x_Ga_1−x_As, In_x_Ga_1−x_Sb and Ga_x_Al_1−x_As nanowires. The reverse behavior is obtained for InSb_x_As_1−x_ nanowires. ^2^ Applied to In_x_Ga_1−x_As nanowires.

**Table 3:** Summary of the liquid–solid incorporation models.

	Equilibrium Model		Nucleation Model		Kinetic Model	
	General Case	Decoupling Similar Results as →	Wilemskii Approach(da/dx=0)	General Case	General Case	$\mathrm{Pure}\;\mathrm{Kinetic}\;(\mu_{AD}\gg 0,$ $\Delta \mu_{BD}\gg 0$
Describes	border between the liquid and solid phases		critical island		fractional monolayer (supercritical island)	
Required supersaturation	zero		low		high	
Governing equation	∆μ=0	$\left\{\begin{array}{c} ∆\mu_{AD}=0\\ ∆\mu_{BD}=0 \end{array}\right.$	$∆\mu_{AD}=∆\mu_{BD}$	$\frac{\partial \Delta \mu }{\partial x}=\Delta \mu \frac{2}{a}\frac{da}{dx}$	$\left\{\begin{array}{c} \frac{dN_{AD}}{dt}=W_{AD}\left(1-e^{\frac{dF}{dN_{AD}}}\right)\\ \frac{dN_{BD}}{dt}=W_{BD}\left(1-e^{\frac{dF}{dN_{BD}}}\right) \end{array}\right.$	$\left\{\begin{array}{c} \frac{dN_{AD}}{dt}=W_{AD}\\ \frac{dN_{BD}}{dt}=W_{BD} \end{array}\right.$
Analytic formula	$x=\frac{1}{1+\frac{\Delta \mu_{AD}}{\Delta \mu_{BD}}}$	$\left\{\begin{array}{c} x=\frac{1}{1+\frac{1-y}{y}e^{2\omega_{S}\left(1/2-x\right)-b}}\\ c_{D}=\frac{x}{y}\frac{1}{c_{tot}}e^{\omega_{S}{\left(1-x\right)}^{2}+b_{D}} \end{array}\right.$	$x=\frac{1}{1+\frac{1-y}{y}e^{2\omega_{S}\left(1/2-x\right)-b}}$	$\frac{\Delta \mu_{AD}}{{\Delta \mu }_{BD}}=1+\frac{\frac{2}{a}\frac{da}{dx}}{1-\frac{2}{a}\frac{da}{dx}x}$	$x=\frac{1}{1+k\frac{\left(1-y\right)}{y}\frac{\left(1-e^{-\Delta \mu_{BD}}\right)}{\left(1-e^{-\Delta \mu_{AD}}\right)}}$ (if da/dx=0, $W_{AD}=K_{AD}c_{A}c_{D}$ and $W_{BD}=K_{BD}c_{B}c_{D}$)	$x=\frac{1}{1+k\frac{1-y}{y}}$ (if $W_{AD}=K_{AD}c_{A}c_{D}$ and $W_{BD}=K_{BD}c_{B}c_{D}$)
Supression of the miscibility gap ^1^	no	no	no	yes	yes	no miscibility gap
Advantages	• gives the fundamental limit	• no free parameters	• almost no effect of $c_{V}$ • closed form approximation	• realistic description	• flexibility • capable to fit experimental data [68]	• one-parameter model
Drawbacks	• infinite nucleation time	• inflexibility • no simple formula • infinite nucleation time	• nanowire composition should repeat the composition of the critical nucleus	• no simple formula • uncertainty in the surface energy values	• high sensitivity of x(y) to $c_{V}$ • unknown temperature effect on $c_{V}$ and $c_{U}$	• disregards thermodynamics
Heterostructures	Not studied	[21,111]	[115,116]	Not studied	[148]	[127]

^1^ At a fixed temperature.

## Data Availability

Not applicable.

## Supplementary Materials

The supporting information can be downloaded at: https://www.mdpi.com/article/10.3390/nano13101659/s1. Table S1: Ternary interaction parameters of the As-Au-Ga-In-Sb system; Table S2: Binary interaction parameters of the As-Au-Ga-In-Sb system; Table S3: The chemical potentials of the species in the solid; Table S4: The chemical potentials of the species in the liquid; Table S5: GHSER. References [152,153,154,155,156,157,158,159,160,161,162,163,164] are cited in the supplementary materials.
