# Supplementary material for: An Overview of Modeling Approaches for Compositional Control in III–V Ternary Nanowires
Source: Nanomaterials (Basel). 2023 May 17;13(10):1659. doi: 10.3390/nano13101659 (PMC10221609; doi:10.3390/nano13101659)
Supplement: Supplementary file 1 [file nanomaterials-13-01659-s001.zip › nanomaterials-2391986-supplementary.pdf]

Supplementary Information

Theory

The difference of the chemical potentials between liquid and solid is given by

$$\Delta\mu = x\Delta\mu_{AD} + (1-x)\Delta\mu_{BD}, \quad \text{(S1)}$$

where the chemical potential differences of AD and BD pairs in the liquid and in the solid state are given by

$$\Delta\mu_{AD} = \mu_A^L + \mu_D^L - \mu_{AD}^s, \quad \text{(S2)}$$

$$\Delta\mu_{BD} = \mu_B^L + \mu_D^L - \mu_{BD}^s. \quad \text{(S3)}$$

The chemical potentials of the species in the liquid are expressed as $\mu_i^L = \mu_i^0 + k_B T ln(c_i) + \vartheta_i$, namely

$$\begin{aligned}\mu_A^L = \mu_A^0 + k_B T ln(c_A) + \omega_{AB}{c_B}^2 + \omega_{AD}{c_D}^2 \quad \text{(S4)}\\ + \omega_{AU}{c_U}^2\\ + (\omega_{AB} + \omega_{AD} - \omega_{BD})c_B c_D\\ + (\omega_{AB} + \omega_{AU} - \omega_{BU})c_B c_U\\ + (\omega_{AD} + \omega_{AU} - \omega_{DU})c_D c_U\\ + (c_B c_D - 2c_A c_B c_D)\omega_{ABD}\\ + (c_B c_U - 2c_A c_B c_U)\omega_{ABU}\\ + (c_D c_U - 2c_A c_D c_U)\omega_{ADU}\\ - 2c_B c_D c_U \omega_{BDU}\\ + (2c_A c_B - {c_B}^2 - 2c_B {c_A}^2\\ + 2{c_B}^2 c_A)\omega'_{AB}\\ + (2c_A c_D - {c_D}^2 - 2c_D {c_A}^2\\ + 2{c_D}^2 c_A)\omega'_{AD}\\ + (2c_A c_U - {c_U}^2 - 2c_U {c_A}^2\\ + 2{c_U}^2 c_A)\omega'_{AU}\\ + 2(c_B {c_D}^2 - {c_B}^2 c_D)\omega'_{BD}\\ + 2(c_B {c_U}^2 - {c_B}^2 c_U)\omega'_{BU}\\ + 2(c_D {c_U}^2 - {c_D}^2 c_U)\omega'_{DU},\end{aligned}$$

$$\begin{aligned}\mu_B^L = \mu_B^0 + k_B T ln(c_B) + \omega_{AB}{c_A}^2 + \omega_{BD}{c_D}^2 \quad \text{(S5)}\\ + \omega_{BU}{c_U}^2\\ + (\omega_{AB} + \omega_{BD} - \omega_{AD})c_A c_D\\ + (\omega_{AB} + \omega_{BU} - \omega_{AU})c_A c_U\\ + (\omega_{BD} + \omega_{BU} - \omega_{DU})c_D c_U\\ + (c_A c_D - 2c_A c_B c_D)\omega_{ABD}\\ + (c_A c_U - 2c_A c_B c_U)\omega_{ABU}\\ + (c_D c_U - 2c_B c_D c_U)\omega_{BDU}\\ - 2c_A c_D c_U \omega_{ADU}\\ + (2c_A c_B - {c_A}^2 - 2c_A {c_B}^2\\ + 2{c_A}^2 c_B)\omega'_{BA}\\ + (2c_B c_D - {c_D}^2 - 2c_D {c_B}^2\\ + 2{c_D}^2 c_B)\omega'_{BD}\\ + (2c_B c_U - {c_U}^2 - 2c_U {c_B}^2\\ + 2{c_U}^2 c_B)\omega'_{BU}\\ + 2(c_A {c_D}^2 - {c_A}^2 c_D)\omega'_{AD}\\ + 2(c_A {c_U}^2 - {c_A}^2 c_U)\omega'_{AU}\\ + 2(c_D {c_U}^2 - {c_D}^2 c_U)\omega'_{DU},\end{aligned}$$

$$\mu_D^L = \mu_D^0 + k_B T ln(c_D) + \omega_{AD} {c_A}^2 + \omega_{BD} {c_B}^2 + \omega_{DU} {c_U}^2 + (\omega_{AD} + \omega_{BD} - \omega_{AB}) c_A c_B + (\omega_{AD} + \omega_{DU} - \omega_{AU}) c_A c_U + (\omega_{BD} + \omega_{DU} - \omega_{BU}) c_B c_U + (c_A c_B - 2 c_A c_B c_D) \omega_{ABD} + (c_A c_U - 2 c_A c_D c_U) \omega_{ADU} + (c_B c_U - 2 c_B c_D c_U) \omega_{BDU} - 2 c_A c_B c_U \omega_{ABU} + (2 c_A c_D - {c_A}^2 - 2 c_A {c_D}^2 + 2 {c_A}^2 c_D) \omega'_{DA} + (2 c_B c_D - {c_B}^2 - 2 c_B {c_D}^2 + 2 {c_B}^2 c_D) \omega'_{DB} + (2 c_D c_U - {c_U}^2 - 2 c_U {c_D}^2 + 2 {c_U}^2 c_D) \omega'_{DU} + 2 (c_A {c_B}^2 - {c_A}^2 c_B) \omega'_{AB} + 2 (c_A {c_U}^2 - {c_A}^2 c_U) \omega'_{AU} + 2 (c_B {c_U}^2 - {c_B}^2 c_U) \omega'_{BU}. \quad (S6)$$

Here μ^0 is the Gibbs free energy of the corresponding pure element, ω_{ij} and ω_{ijk} are the binary and ternary interaction parameters.

The chemical potentials in the solid of AD and BD binary species are given by

$$\mu_{AD}^s = \mu_{AD}^0 + k_B T ln x + (1-x)^2 [\omega_S + (4x-1)\omega'_S], \quad (S7)$$

$$\mu_{BD}^s = \mu_{BD}^0 + k_B T ln(1-x) + x^2 [\omega_S + (4x-3)\omega'_S]. \quad (S8)$$

Here ω_S and ω'_S are the temperature dependent interaction parameters of zeroth and first order, and μ^0 is the Gibbs free energy of the corresponding binary compound.

The parameter b is given by [1]

$$b = \alpha - 6 c_{tot}^2 \omega'_{AB} - 2 c_{tot} [\omega_{AB} - 3 c_{tot} \omega'_{AB} - c_D (\omega'_{AD} + \omega'_{BD}) - c_U (\omega'_{AU} + \omega'_{BU}) + c_D \omega_{ABD} + c_U \omega_{ABU}] \quad (S9)$$

with

$$\alpha = \Delta\mu_{AD}^0 - \Delta\mu_{BD}^0 + (\omega_{AB} - c_{tot} \omega'_{AB} - 2 c_D \omega'_{BD} - 2 c_U \omega'_{BU} + c_D \omega_{ABD} + c_U \omega_{ABU}) c_{tot} + (\omega_{AD} - \omega_{BD}) c_D + (\omega_{AU} - \omega_{BU}) c_U - (\omega'_{AD} - \omega'_{BD}) c_D^2 - (\omega'_{AU} - \omega'_{BU}) c_U^2 + (\omega_{ADU} - \omega_{BDU}) c_D c_U. \quad (S10)$$

The parameter b_D is given by

$$b_D = \mu_{AD}^0 - \mu_A^0 - \mu_D^0 - \vartheta_A - \vartheta_D. \quad (S11)$$

Parameters for As-Ga-In, In-Ga-Sb and Al-Ga-As material systems

Table S1. Ternary interaction parameters of the As-Au-Ga-In-Sb system. The energy values are in J/mole and the temperature is in K.

Ternary System	ω_{ijk}	ω_s	Refs.
Al-As-Ga	-55508.5 + 33.2087·T	2187	[2]
Ga-In-Sb	-5072.76 - 10.8842·T	9093 - 2.8698·T	[3]
In-Ga-As	-12889.5	19698.8 - 7.51693·T	[4]
In-Ga-Au	20500		[5]
Au-In-Sb	1129.0976 - 16.232·T		[6]

Table S2. Binary interaction parameters of the As-Au-Ga-In-Sb system. The energy values are in J/mole and the temperature is in K.

Binary Systems	ω_{ij}	ω'_{ij}	Refs.
Al-Ga	2613.3 - 2.94533·T	692.4 - 0.09271·T	[7]
Al-As	-15693 - 34.163·T		[7]
As-Ga	-25503.6 - 4.3109·T	-5174.7	[7]
Ga-Sb	-13953.8 + 71.07866·T - 9.6232·T·ln(T)	1722.9 - 1.92588·T	[7]
As-In	-15851 - 11.27053·T	-1219.5	[7]
In-Sb	-25631.2 + 102.9324·T - 13.45816·T·ln(T)	-2115.4 - 1.31907·T	[7]
Ga-In	4450 + 1.19185·T	0.25943	[8]
Al-Au	-131996.19 + 36.42·T	40781.83 - 1.896·T	[9]
Au-Sb	-10288.0428 - 14.7865028·T	-2901.66787 - 7.2503632·T	[10]
As-Au	18160.759 - 14.327686·T	-13132.906 + 13.229781·T	[11]
Au-In	-76196.19 + 64.2914·T - 6.6375·T·ln(T)	-31134.02 + 81.3582·T - 8.5134·T·ln(T)	[12]
Au-Ga	-71830.123 + 42.286·T - 4.289·T·ln(T)	-22892.323 + 5.069·T	[13]

Table S3. The chemical potentials of the species in the solid. The energy values are in J/mole and the temperature is in K.

Binary Systems	μ^0_{ij}	Refs.
Al-As	-117130+7.78·T + $GHSER_{Al}$ + $GHSER_{As}$	[7]
Ga-As	-104352 + 265.43256·T - 48.681258·T·ln(T) - 11.158E-04·T^2 + 127670·T^{-1} - 7.1378E-07·T^3 (2)	[7]
In-As	-73057.2 + 230.91896·T - 45.187942·T·ln(T) - 0.00773·T^2 + 69438·T^{-1} + 14.18E-08·T^3	[7]
Ga-Sb	-43476.2 - 21.07528·T + 5.385752·T·ln(T) - 0.00275582·T^2 + $GHSER_{Ga}$+ $GHSER_{Sb}$	[7]
In-Sb	-31698.6 + 0.586278·T + 2.587162·T·ln (T) + $GHSER_{In}$ + $GHSER_{Sb}$	[7]

Table S4. The chemical potentials of the species in the liquid. The energy values are in J/mole and the temperature is in K.

Systems	μ^0_i	Refs.
Al	(298.15<T<700) 3028.879 + 125.251171·T - 24.3671976·T·ln(T) - 0.001884662·T^2 - 8.77664E-07·T^3 + 74092·T^{-1} + 7.9337E-20·T^7<br>(700<T<933.47) - 271.21 + 211.206579·T - 38.5844296·T·ln(T) + 0.018531982·T^2 - 5.764227E-06·T^3 + 74092·T^{-1} + 7.9337E-20·T^7	[14]
As	(298.15<T<1200) 24442.9 - 22.424679·T + $GHSER_{As}$	[14]
Ga	(200<T<302.91) 5491.298-18.073995·T + $GHSER_{Ga}$ - 7.0171E-17·T^7<br>(302.91<T<4000) 5666.455-18.681147·T + $GHSER_{Ga}$ - 1.64547E+23·T^{-9}	[14]
In	(298.15<T<429.75) -3696.798 + 84.701255·T - 21.8386·T·ln(T) - 0.00572566·T^2 - 2.120321E-06·T^3 - 22906·T^{-1} - 5.59058E-20·T^7<br>(429.75<T<3800) - 3749.81 + 116.835784·T - 27.4562·T·ln(T) + 5.4607E-04·T^2 - 8.367E-08·T^3 - 211708·T^{-1}	[14]
Sb	(298.15<T<903.78) 19822.328 - 21.923164·T + $GHSER_{Sb}$ - 1.74847E-20·T^7<br>(903.78<T<2000) 8175.359 + 147.455986·T - 31.38·T·ln(T)	[14]

Table S5. GHSER. The energy values are in J/mole and the temperature is in K.

Systems	$GHSER_i$	Refs.
Al	$(298.15<T<700)\ -7976.15 + 137.093038\cdot T - 24.3671976\cdot T\cdot \ln(T)$<br>$- 0.001884662\cdot T^{2} - 8.77664E\text{-}07\cdot T^{3} + 74092\cdot T^{-1}$<br>$(700<T<933.47)\ -11276.24 + 223.048446\cdot T - 8.5844296\cdot T\cdot \ln(T)$<br>$+ 0.018531982\cdot T^{2} - 5.764227E\text{-}06\cdot T^{3} + 74092\cdot T^{-1}$	[14]
As	$(298.15<T<1090)\ -7270.447 + 122.211069\cdot T - 23.3144\cdot T\cdot \ln(T)$<br>$- 0.00271613\cdot T^{2} + 11600\cdot T^{-1}$<br>$(1090<T<1200)\ -10454.913 + 163.457433\cdot T - 29.216037\cdot T\cdot \ln(T)$	[14]
Ga	$(298.15<T<302.91)\ -21312.331 + 585.263691\cdot T - 108.228783\cdot T\cdot \ln(T)$<br>$+ 0.227155636\cdot T^{2} - 1.18575257E\text{-}04\cdot T^{3} + 439954\cdot T^{-1}$<br>$(302.91<T<4000)\ -7055.643 + 132.73019\cdot T - 26.0692906\cdot T\cdot \ln(T)$<br>$+ 1.506E\text{-}04\cdot T^{2} - 4.0173E\text{-}08\cdot T^{3} - 118332\cdot T^{-1} + 1.64547E{+}23\cdot T^{-9}$	[14]
In	$(298.15<T<429.75)\ -6978.89 + 92.338115\cdot T - 21.8386\cdot T\cdot \ln(T)$<br>$- 0.00572566\cdot T^{2} - 2.120321E\text{-}06\cdot T^{3} - 22906\cdot T^{-1}$<br>$(429.75<T<3800)\ -7033.516 + 124.476588\cdot T - 27.4562\cdot T\cdot \ln(T)$<br>$+ 5.4607E\text{-}04\cdot T^{2} - 8.367E\text{-}08\cdot T^{3} - 211708\cdot T^{-1} + 3.53116E{+}22\cdot T^{-9}$	[14]
Sb	$(298.15<T<903.78)\ -9242.858 + 156.154689\cdot T - 30.5130752\cdot T\cdot \ln(T)$<br>$+ 0.007748768\cdot T^{2} - 3.003415E\text{-}06\cdot T^{3} + 100625\cdot T^{-1}$<br>$(903.78<T<2000)\ -11738.83 + 169.485872\cdot T - 31.38\cdot T\cdot \ln(T) + 1.616849E{+}27\cdot T^{-9}$	[14]

References

1. Leshchenko, E.D.; Ghasemi, M.; Dubrovskii, V.G.; Johansson, J. Nucleation-limited composition of ternary III–V nanowires forming from quaternary gold based liquid alloys. *CrystEngComm* **2018**, *20*, 1649-1655.
2. Li, C.; Li, J.B.; Du, Z.; Lu, L.; Zhang, W. A thermodynamic reassessment of the Al-As-Ga system. *Journal of Phase Equilibria* **2001**, *22*, 26-33.
3. Yang, J.; Watson, A. An assessment of phase diagram and thermodynamic properties of the gallium-1ndium-antimony system. *Calphad* **1994**, *18*, 165-175.
4. Shen, J.-Y.; Chatillon, C.; Ansara, I.; Watson, A.; Rugg, B.; Chart, T. Optimisation of the thermodynamic and phase diagram data in the ternary As-Ga-In system. *Calphad* **1995**, *19*, 215-226.
5. Ghasemi, M.; Sundman, B.; Fries, S.G.; Johansson, J. The thermodynamic assessment of the Au–In–Ga system. *Journal of Alloys and Compounds* **2014**, *600*, 178-185.
6. Gomidzelovic, L.; Zivkovic, D.; Mihajlovic, I.; Trujic, V. Predicting of thermodynamics properties of ternary Au-In-Sb system. *Archives of metallurgy and materials* **2006**, *51*, 355-363.
7. Ansara, I.; Chatillon, C.; Lukas, H.L.; Nishizawa, T.; Ohtani, H.; Ishida, K.; Hillert, M.; Sundman, B.; Argent, B.B.; Watson, A.; et al. A binary database for III–V compound semiconductor systems. *Calphad* **1994**, *18*, 177-222.
8. Anderson, T.J.; Ansara, I. The Ga-In (Gallium-Indium) System. *Journal of Phase Equilibria* **2007**, *12*, 64-72.
9. Li, M.; Li, C.; Wang, F.; Luo, D.; Zhang, W. Thermodynamic assessment of the Al–Au system. *Journal of Alloys and Compounds* **2004**, *385*, 199-206.
10. Kim, J.H.; Jeong, S.W.; Lee, H.M. A thermodynamic study of phase equilibria in the Au-Sb-Sn solder system. *Journal of Electronic Materials* **2002**, *31*, 557-563.
11. Ghasemi, M.; Johansson, J. Phase diagrams for understanding gold-seeded growth of GaAs and InAs nanowires. *Journal of Physics D: Applied Physics* **2017**, *50*, 134002.
12. Liu, H.S.; Cui, Y.; Ishida, K.; Jin, Z.P. Thermodynamic reassessment of the Au◎In binary system. *Calphad* **2003**, *27*, 27-37.
13. Wang, J.; Liu, Y.J.; Liu, L.B.; Zhou, H.Y.; Jin, Z.P. Thermodynamic assessment of the Au–Ga binary system. *Calphad* **2011**, *35*, 242-248.
14. Dinsdale, A.T. SGTE data for pure elements. *Calphad* **1991**, *15*, 317-425.
